# Real-Time Monitoring of the Level and Activity of Intracellular Glutathione in Live Cells at Atomic Resolution by ^19^F-NMR

**DOI:** 10.1021/acscentsci.3c00385

**Published:** 2023-07-21

**Authors:** Chao-Yu Cui, Bin Li, Xun-Cheng Su

**Affiliations:** State Key Laboratory of Elemento-organic Chemistry, College of Chemistry, Nankai University, Tianjin 300071, China

## Abstract

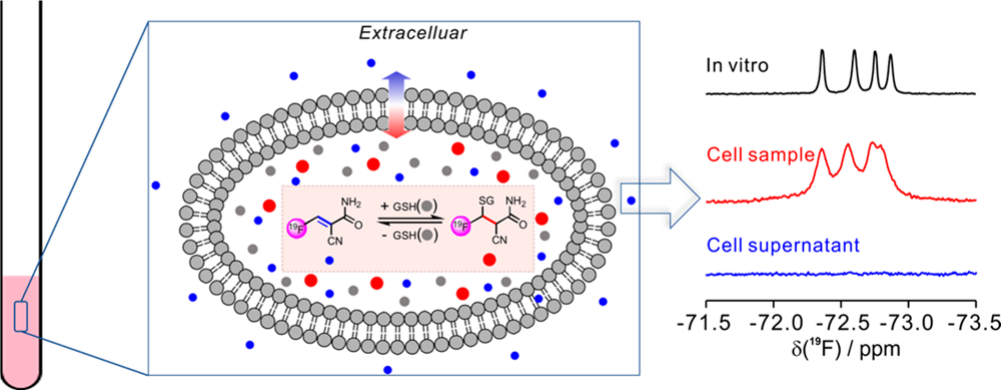

Visualization and quantification of important biomolecules like glutathione (GSH) in live cells are highly important. The existing methods are mostly from optical detection and lack of atomic resolution on the activity of GSH. Here, we present a sensitive ^19^F-NMR method to quantify real-time variations of GSH in live cells in a reversible manner. This NMR method prevents extracellular leakage and irreversible consumption of intracellular GSH during the detection. The high performance of the reactive ^19^F-probe enables accurate determination of intracellular GSH content at atomic resolution, from which information on GSH variations with respect to the extracellular and intracellular conditions can be inferred. In addition, we demonstrate the applicability of this NMR method to quantify the GSH levels between different live cell lines and to disclose the distinct differences between the intracellular environment and cell lysates. We foresee the application of ^19^F-NMR to monitor real-time variations of intracellular GSH levels in relation to GSH-involved central cellular processes.

## Abstract 2

A reversible ^19^F-probe demonstrates an efficient way to quantify glutathione (GSH) levels and real-time monitor variations of GSH in live cells by NMR spectrometry.

## Introduction

Glutathione (GSH) is the most abundant nonprotein thiol in living organisms and has multiple important functions.^1−4^ GSH generally exists in two forms: reduced glutathione (GSH) and oxidized glutathione (GSSG). Under physiological conditions, the reduced GSH is the major form, with its concentration from 10 to 100-fold higher than the oxidized one.^5^ GSH is able to scavenge reactive oxygen and nitrogen species, thereby contributing to the control of redox homeostasis. The homeostasis of intracellular GSH is tightly controlled by intracellular enzymes,^3^ and the increased GSH level in tumor cells is often associated with increased resistance to cancer chemotherapeutic drugs.^6^ Meanwhile, the thiol group in GSH is an excellent coordinating atom for intracellular transition metal ions, including copper, iron, and zinc, which are essential for living systems, and forms stable metal complexes in cells.^7,8^ In addition, GSH plays important roles in drug transportation and detoxification,^9^ and recent findings suggest that the efflux of GSH is an unexpected regulator of ferroptosis sensitivity in cells.^10^

Real-time quantification of intracellular GSH is insightful for monitoring the variations of intracellular physiological conditions in relation to pathophysiological and metabolic processes. Most methods rely on the irreversible consumption of GSH to detect changes of the detecting probes either in spectral or imaging density, which are unable to monitor the real-time changes of GSH levels in live cells.^11,12^ This irreversible detection strategy introduces the assumption that GSH can be produced continuously from live cells, which shifts the homeostasis of intracellular GSH under basal conditions. To overcome this limitation, a reversible detection concept was proposed,^13,14^ and the detecting rationale relies on the equilibrium reaction between the fluorescent probe and GSH in the formation of an unstable adduct, which has a different fluorescent spectrum from the free probe. The widely used reversible probes contain either a Michael receptor or nucleophilic substitution core for free thiols.^15−18^ Both irreversible and reversible approaches have made remarkable progress in detecting intracellular GSH.^13,14,19,20^ Recent evidence indicates remarkable variations of GSH stability are present in different cell lines, and the hydrolysis of GSH produces Cys-Gly and Cys, in which each contains a reactive free thiol group, and both are prone to perturb the GSH quantification.^21^ An additional concern is the impact of the nonspecific association of a hydrophobic fluorophore with the intracellular organelles or membrane on the translocation of probes in cells. Overall, residual specific or atomic level information about the GSH homeostasis in live cells remains unresolved.

NMR is a noninvasive method that can provide rich structural information at the atomic-level resolution of biomolecules and offer a wide range of time-scale dynamics for the biological molecules under physiological conditions. Especially, in-cell NMR has offered an opportunity to study the protein structure and dynamics in situ or in live cells.^22−25^^19^F-NMR has the advantage of high sensitivity, large range of chemical shift, and no-background interference of intracellular components, and is well suitable for biological assay.^26−30^ Inspired by the reversible detection of intracellular GSH by fluorescence probes proposed by the earlier reports,^13,14^ and 2D NMR assay of selectively isotope labeled GSH and protein samples in live cells,^31,32^ we attempted to establish a ^19^F-NMR method of monitoring the GSH level in live cells via a reversible manner with respect to the real-time cell response to the variations of the growing media.

## Results and Discussion

### Design of Reversible ^19^F Probe and Structure Optimization

**Figure 1:**
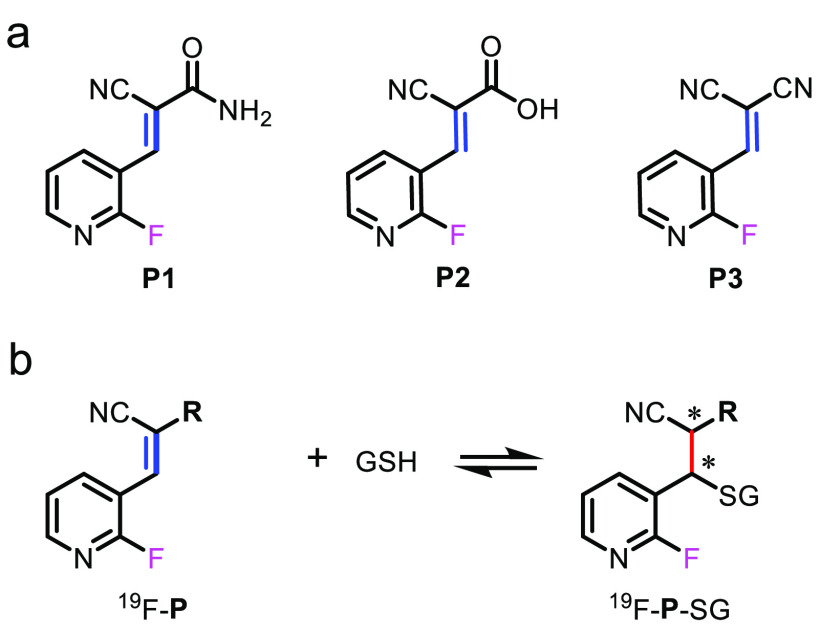
(a) Chemical structures of ^19^F-probes for reversible in-cell GSH quantification. (b) Reversible chemical reaction of ^19^F-probe and GSH under physiological conditions, in which the new chiral center produced in this reaction was labeled with a star.

Several probes that each contain a thiol-reactive moiety, Michael receptor, and sensitive ^19^F-reporter were designed and synthesized (Figure 1a and Supporting Information). The performance of the ^19^F-probes in the reversible detection of GSH (Figure 1b) and the applications in monitoring GSH levels in live cells were assessed accordingly.

The thiol–ene Michael addition reaction is widely used in protein bioconjugation,^33^ and the α,β-unsaturated organic molecules stand out with their unique tunable reactivity from irreversible to reversible with respect to the electron withdrawing capacity of the substituent at the α-position in the α,β-unsaturated site.^17,34−36^ To achieve high sensitivity of ^19^F to the chemical environment changes of the ^19^F-probe in reaction with the target analytes, a ^19^F-reporter is anchored in the *ortho*-position of the reaction center in the pyridine, which is water soluble. To refrain from the large and multiple J couplings of fluorine with vicinal protons and to have better sensitivity to the variations of chemical surroundings, one single F rather than a CF_3_ is anchored in a position that has no high density of protons in pyridine (Figure 1a). To have a reversible reaction with free thiols, a cyano group was incorporated in the α-position of the α,β-unsaturated probe containing a ^19^F-reporter. Following this notion, the α,β-unsaturated ^19^F-probes, each containing both F and cyano, were designed and synthesized (Figure 1, Figures S1–S3 and Supporting Information). The ^1^H NMR and ^1^H-^1^H NOESY spectra of the three probes were recorded to confirm the configuration (Figures S1–S3), and the NMR data showed that these ^19^F-probes present a dominant *E*-configuration in chloroform and aqueous solution (Figure 1a).

**Figure 2:**
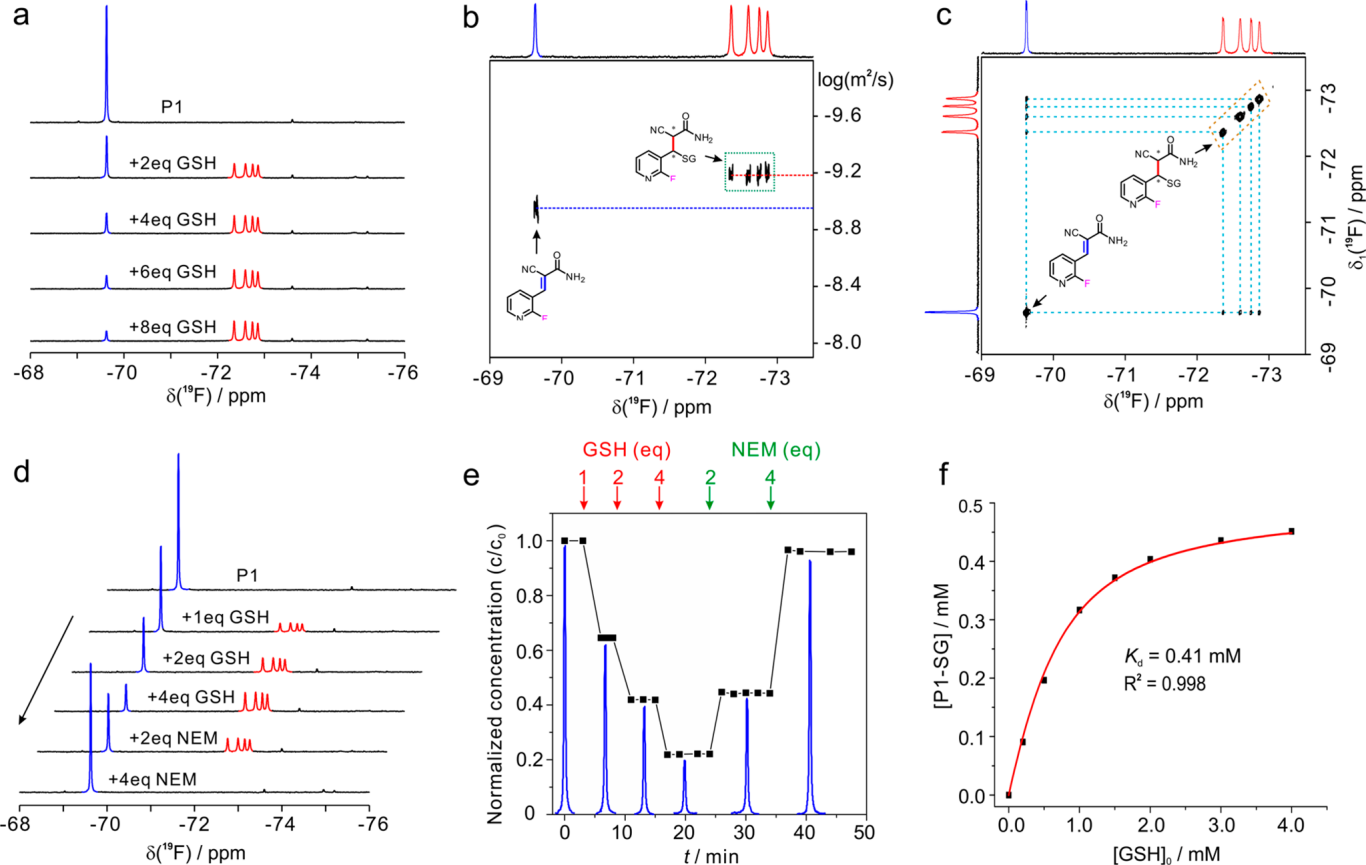
Reactivity assay of ^19^F-probe with GSH and reversibility evaluation of the adduct. (a) ^19^F-NMR spectra of 0.5 mM **P1** probe with the addition of GSH. (b) 2D DOSY ^19^F-NMR spectrum recorded for the mixture of 0.5 mM **P1** and 2.0 mM GSH in 20 mM PB buffer at pH 7.5. (c) 2D EXSY ^19^F-NMR spectrum recorded for the solution of 1.0 mM **P1** and 2.0 mM GSH with a mixing time of 70 ms. (d) 1D ^19^F-NMR spectra recorded for the sample of 0.5 mM **P1** with the stepwise addition of GSH and then *N*-ethylmaleimide (NEM). (e) The normalized NMR peak integral area change of free **P1** with respect to the addition of GSH and then NEM as shown in (d). (f) Correlation curve of [**P1**-SG] versus GSH, and determination of dissociation constant formed by **P1** and GSH, **P1**-SG. The peaks marked in blue are free **P1**, and the peaks marked in red are the **P1**-SG adduct. The spectra were recorded in 20 mM phosphate buffer at pH 7.5 and at 298 K with a ^1^H 800 MHz NMR spectrometer.

Because either the hydrolysis of Michael receptor or slow reaction rates in the formation and dissociation of the thiol adducts compromises the thiol quantification, we first evaluated the stability of these Michael receptors in aqueous solution and the kinetic properties toward free thiols. The results indicated that the three ^19^F-probes vary greatly in stability close to the physiological conditions. For example, **P1** and **P2** are relatively stable (Figure S4 and Table S1), but **P3** is the least stable and hydrolyzes completely within a couple of hours in 20 mM phosphate buffer at pH 7.5. **P1** displays gradual hydrolysis in solution and has a half-lifetime over 18 h under physiological conditions, and **P2** is more stable and has no significant hydrolysis under the above conditions. **P1** and **P2** were selected for testing the reactivity with small biothiols, and distinct chemical shift profiles were determined in the interactions. **P1** reacts fast with GSH, which is readily monitored by 1D NMR spectra (Figure 2a). The reaction completes within 20 s as determined by the UV–visible spectrometry, which is sufficiently fast for quantification of GSH by the NMR method. In contrast, the reaction of **P2** and GSH proceeds in a rather slower manner, and it takes over 30 min to reach equilibrium (Figure S5). Considering the above evaluations, we used **P1** to testify to the GSH quantification in the following assay.

### High Performance of Reversible ^19^F-Probe in GSH Quantification

To testify the feasibility of biothiol quantification in live cells by the reactive ^19^F-probe, we first assessed the ^19^F chemical shift profile of the ^19^F-probe with reaction to GSH. The **P1**-SG adduct presents four fluorine NMR signals with similarly equivalent populations, and these new signals are well distinguished from that of free **P1** (Figure 2a). Because two chiral centers are generated upon formation of the **P1**-SG adduct, four chiral products (*R*, *R*; *R*, *S*; S, *R*; *S*, *S*) with respect to the α,β-unsaturated carbon atoms are assumed to be produced, and these chiral products would be distinguishable in the ^19^F-NMR spectra.^37^ The similar population of the four chiral species in solution suggests no chiral selectivity in this reaction (Figure 2a). These four new peaks have similar diffusion coefficients that are significantly different from those of free **P1** as shown in the 2D diffusion ordered spectroscopy (DOSY) experiment (Figure 2b). The molecular mass of the **P1**-SG adduct was also confirmed by mass spectrometry (Figure S6).

The reaction of **P1** and GSH is indeed reversible in aqueous solution. The free **P1** and **P1**-SG coexist in the reaction solution, and the **P1**-SG adduct increases with the concentration of GSH or **P1**. The kinetic property of the **P1**-SG adduct was characterized, and a 2D ^19^F–^19^F EXSY experiment presents four exchange cross peaks with the free **P1** at a mixing time of 70 ms (Figure 2c). The equilibrium is tunable by an irreversible thiol scavenger. Addition of the commonly used *N*-ethylmaleimide (NEM) regenerates free **P1** with a decrease of the **P1**-SG adduct, and no other NMR signals are determined (Figure 2d,e). In the detecting solution, the concentration of **P1** and GSH formed adduct, [**P1**-SG], and free **P1**, [**P1**], is proportional to the integral area of the NMR signal in the ^19^F-NMR spectra. There is an excellent correlation between [**P1**-SG] and [GSH]_0_(*R*^2^ = 0.998) (Figure 2f). Overall, the quantitative regeneration of free **P1** in a mixture of **P1** and GSH supports the notion of monitoring the variation of intracellular GSH levels by NMR.

### Selectivity of **P1** to Intracellular Nucleophiles and Biothiols

**Figure 3:**
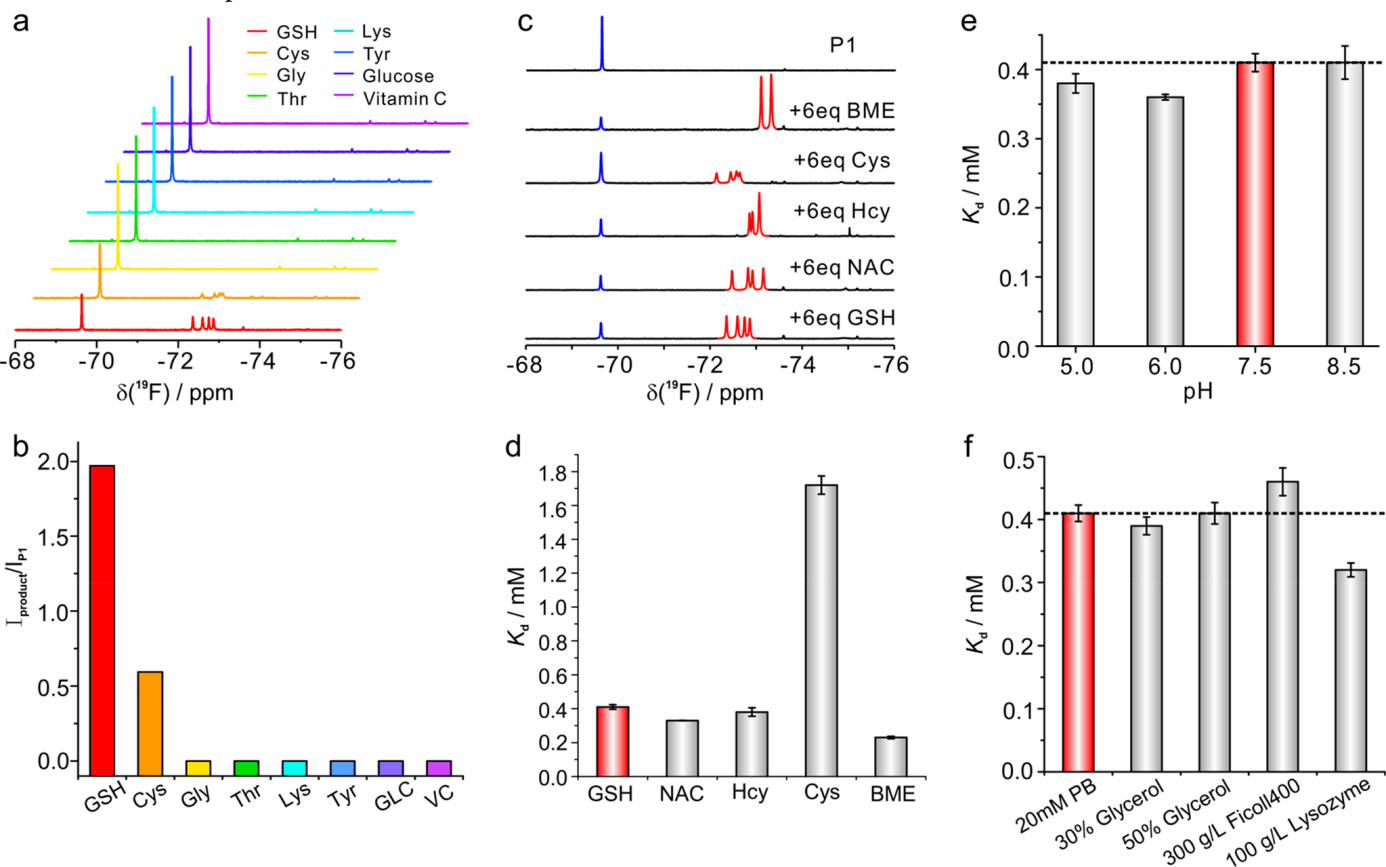
Selectivity assay of ^19^F-probe toward GSH under different conditions and evaluation of the stability of **P1**-SG adduct formed by the ^19^F-probe and GSH in different crowding media and at different pHs. (a) ^19^F-NMR spectra recorded for the mixture of 0.5 mM **P1** after incubation with 1 mM GSH, 1 mM amino acid (Cys, Gly, Thr, Lys, or Tyr), 1 mM glucose (GLC), and 1 mM vitamin C (VC) for 1 h, respectively. (b) The integral area ratio of the Michael addition adducts of **P1** and free **P1** upon addition of GSH and other small molecules as shown in (a), in which only GSH and Cys interact with **P1**. (c) Comparison of the 1D ^19^F-NMR spectra recorded for 0.5 mM **P1** upon the addition of 3.0 mM individual biothiols. The NMR signals of free **P1** and its respective biothiol adduct are highlighted in blue and red, respectively. (d) *K*_d_ of **P1** with different biothiol adduct complexes formed in 20 mM PB buffer at pH 7.5. (e) *K*_d_ of **P1**-SG adduct formed by **P1** and GSH determined at different pHs in 20 mM PB buffer. (f) *K*_d_ of **P1**-SG adduct formed by **P1** and GSH determined in buffer and different crowding media with 20 mM PB, at pH 7.5. All the NMR spectra were recorded at 298 K.

We next assessed the selectivity of the ^19^F-probe to common biothiols and other intracellular nucleophiles. As shown in Figure 3, the ^19^F-probe has no obvious interactions with other amino acids (except cysteine), glucose, and vitamin C. High concentrations of amino acids and glucose have no impact on the GSH quantifications (Figure 3b).

Similar to GSH, small biothiols including Cys, homocysteine (Hcy), *N*-acetyl cysteine (NAC), and β-mercaptoethanol (BME) are able to proceed with the Michael addition reactions, but the formed thiol adducts have distinct NMR profiles that are well resolved in the ^19^F-NMR spectra (Figure 3c). The determined dissociation equilibrium constant (*K*_d_) of **P1** with the small thiol adduct increases from 0.23 to 1.72 mM, in which Cys forms the least stable **P1**-Cys adduct complex. The *K*_d_ of **P1-SG** is 0.41 mM, which is similar to those of other biothiol adducts (Figure 3d and Figure S7 and Table S2). Since the concentration of free Cys is usually below 100 μM in live cells and no other small biothiols are detectable in the mammalian cells by NMR,^21^ the low abundance of intracellular Cys and much greater *K*_d_ of its **P1** adduct (1.72 mM) do not impact the accuracy of intracellular GSH quantification. Further, to mimic the intracellular crowding conditions, the *K*_d_ of **P1** and GSH formed adduct was assessed in different crowding media and pH values (Figure 3e,f and Table S3). These experiments showed that the *K*_d_ of **P1**-SG adduct does not vary significantly at different pHs and remains mostly unchanged in different crowding media except for hen egg white lysozyme that binds with **P1** to a certain degree, which affects the stability of the **P1**-SG adduct.

### Evaluation of the Dissociation Constant of P-SG Adduct for Reversible In-Cell NMR Assay

For a reversible reaction, the formation constant of probe-SG adduct is a key factor for the quantification of intracellular GSH because it determines the fraction ratio between free probe and its GSH adduct so that each can be quantified by the spectral methods. Compared with other techniques, NMR has advantages in high resolution and provides atomic-level information on target molecules close to physiological conditions, but it has a shortfall in the low sensitivity range. Therefore, the populations of free ^19^F-probe and its GSH adduct both have to be above the detection limit, generally higher than 5 μM in the NMR spectra for common molecules. The intracellular GSH concentration is generally in a range of 1–10 mM; however, the averaged or bulk concentration of GSH in the detecting NMR tube (a detecting volume of about 150 μL for a 3 mm NMR tube) is greatly decreased to be about 0.2–1.0 mM for the sample containing ∼10^6^ to 10^7^ cells. To restrain the toxicity of ^19^F-probe for the live cells and ensure the concentration of detecting signals above the NMR detectable limit, the sub- to mM range of the ^19^F-probe has to be applied for an in-cell assay. Taken above, the correlation of total GSH concentration and the ratio of probe-SG adduct to the total concentration of ^19^F-probe was assessed with different *K*_d_ values (Figure S8). The *K*_d_ of the probe-SG adduct in a range of 0.1 to 1 mM for the bulk concentration of GSH between 0.2 and 1.0 mM is suitable for accurate NMR measurement within a few minutes per single 1D experiment. Under such conditions, the fractions of free ^19^F-probe and its GSH adduct are both measurable by NMR. Compared with a reversible fluorescent assay,^13^ the appropriate *K*_d_ value for the NMR assay is generally smaller, which is probably due to the higher sensitivity of fluorescence and difference of fluorescent intensity between free probe and its GSH adduct as the NMR assay is generally dependent on the fraction of nuclei spin of interest.

### Quantifying GSH Levels in Different Mammalian Cells

**Figure 4:**
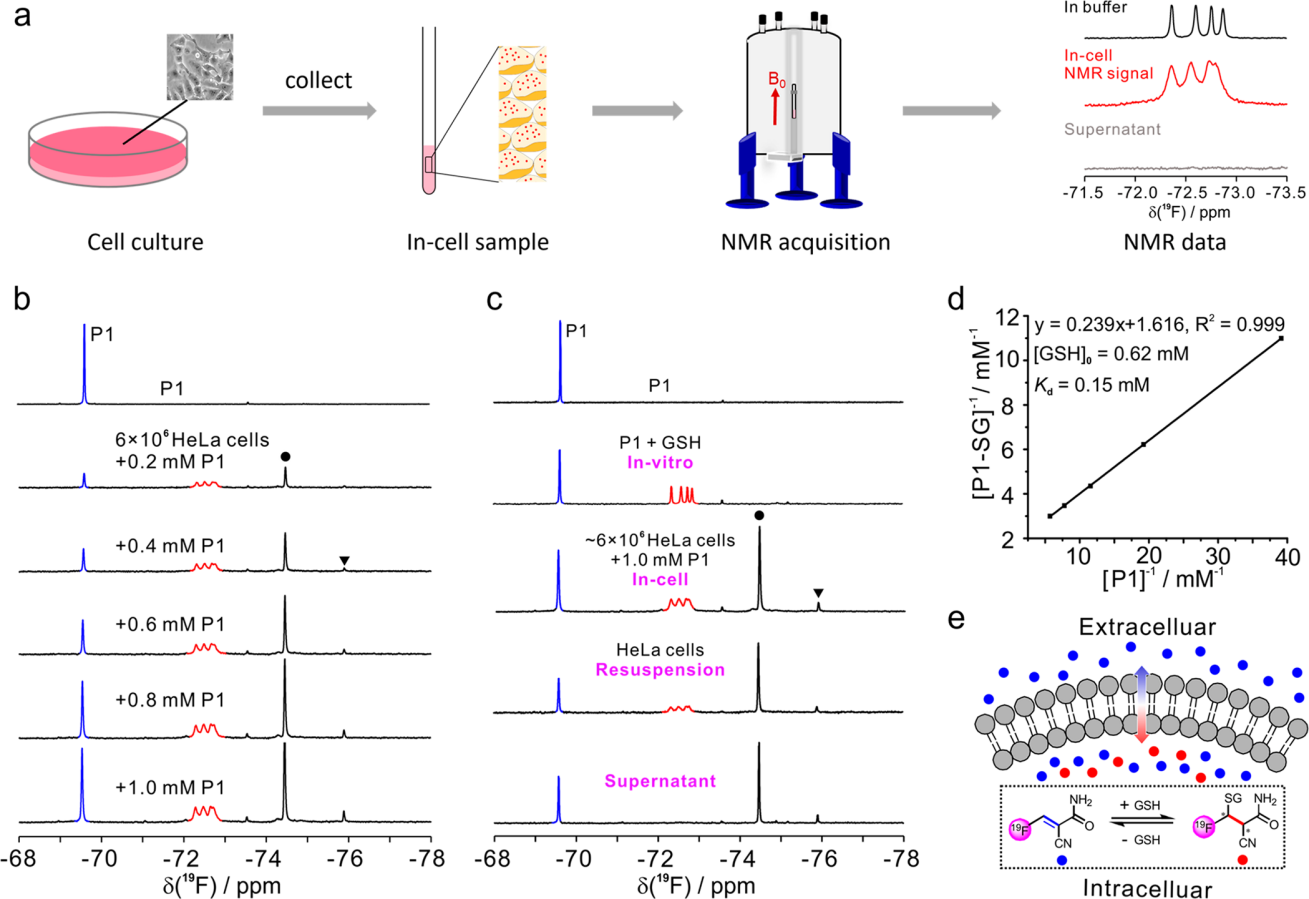
In-cell GSH quantification by the reversible ^19^F-NMR method. (a) Scheme of the GSH quantification in live mammalian cells by NMR. (b) ^19^F-NMR spectra recorded for the sample of HeLa cells with an increase of ^19^F-probe, **P1**. The in-cell NMR signals of GSH and the **P1** adduct are highlighted in red, and NMR signals labeled with solid circles and triangles are the reduction products of free **P1** and aldehyde from the hydrolysis of **P1**, respectively. The top panel is the NMR spectrum of free **P1** in buffer for better comparison. (c) Comparison of the ^19^F-NMR spectra recorded for the samples of HeLa cells from the top to the bottom: free **P1** in buffer; free **P1** with GSH in buffer; HeLa cells after incubation with **P1** (reaction mixture); the resuspended cells of the above reaction mixture; the cell supernatant of the above reaction mixture. (d) Excellent linear correlation between the [**P1**-SG]^−1^ and [**P1**]^−1^ determined in HeLa cells for calculation of *K*_d_ and intracellular GSH levels, in which [**P1**-SG] and [**P1**] are the concentration of the **P1**-SG adduct and free **P1** under equilibrium conditions. (e) The mechanism of intracellular GSH detection with ^19^F-probe, **P1**, which prevents the leak of free GSH into an extracellular environment. The NMR spectra were recorded at 298 K on an ^1^H 800 MHz NMR spectrometer.

The small size, neutrality, high stability (*t*_1/2_ = 18 h), appropriate *K*_d_ of its GSH adduct (0.41 mM), and fast reaction rate with GSH (within seconds) encouraged us to evaluate the performance of **P1** in monitoring the dynamic GSH variations in live cells by ^19^F-NMR. The strategy of tracking atomic-resolution activity of GSH in live cells is outlined in Figure 4a. We noted that incubation of cells with 1 mM **P1** at room temperature up to 24 h has no obvious cell death (about 93% live cells; Figure S9) as assessed by Trypan blue staining, suggesting no significant impact of **P1** on the viability of cells. To minimize the heterogeneity of the live cell samples and to prevent the cell precipitate, we used a 3 mm NMR tube for the live cell assay, which is able to maintain the uniformity of the cell mixture. In general, the cultured cells (∼6 × 10^6^ cells) were resuspended in 150 μL of DMEM buffer and then transferred into a 3 mm NMR tube followed by NMR measurement, and one 1D ^19^F-NMR spectrum was generally completed within 30 min with respect to the original concentration of **P1** (0.2 to 1.0 mM). After addition of **P1** into the live cell mixture, new reactions readily proceed in the live cell samples, and a number of new ^19^F-NMR signals are produced at 298 K (Figure 4b). The NMR signals produced in the live cell samples are overall broader but have chemical shift profiles similar to those of the mixture of **P1** and GSH under in vitro conditions, whereas some additional peaks are produced as marked in labels (Figure 4b,c). Only one NMR signal of free **P1** was determined in the sample of cell mixture, and it has a single broader peak (half line width about 27 Hz) than that of free **P1** in the normal NMR buffer (two peaks separated by ^4^*J*_H–F_ ≈ 9.3 Hz). Since the small molecules generally cross the cell membrane by diffusion,^38^**P1** is small and neutral, and it is expected that it crosses the cell membrane via a diffusion mechanism but in a very fast manner, which is beyond the NMR detection limit. Notably, addition of **P1** into the cell mixture immediately forms the **P1**-SG adduct, and the intracellular **P1-SG** level remains constant after first addition of **P1** over 1 h (Figure S10).

To confirm the NMR signals either from extracellular media or in cells, the cells and the supernatant in the NMR tube were separated and subjected to additional NMR measurements, respectively. As shown in Figure 4c, the recollected cells produced a similar NMR profile as the mixture of **P1** and live cells but with decreased intensity due to the loss of free probe and some fractions of cells during the recollection (Figure 4c). We note that no NMR signals of the **P1**-SG adduct were observed in the supernatant of live cells except of those of free ^19^F-probe and the additional peaks (to be discussed later) (Figure 4c), suggesting that the signals of the **P1**-SG adduct were only present in cells. In addition, no obvious hydrolysis of GSH was determined with a reversible **P1** probe. This is because any association with **P1** would result in chemical shift changes that can be readily identified in the NMR spectra. These data demonstrate that **P1** readily crosses the cell membrane and forms the **P1**-SG adduct with intracellular GSH, and it is also readily excluded from the cell. The signal of **P1** in the live cell sample was the averaged peak between the in cell and extracellular media due to fast exchange. These data indicate that no free GSH is leaked to extracellular media during the NMR detection, which is in great contrast to the irreversible ^19^F-probe for GSH quantification.^21^

The two new additional NMR signals with chemical shifts of −74.45 and −75.89 ppm, respectively, are absent from the *in vitro* samples (Figure 4b,c), and they are gradually produced in the live cells from **P1**. These two signals are readily eluted outside of cells as free **P1**. We assigned the NMR signal at −74.45 ppm to the reduction of the olefinic bond in the free ^19^F-probe, which is identical to the model product of reduced **P1** by organic synthesis (Figure S11). In addition, the NMR signal at −75.89 ppm from the intracellular species, which represents a very low fraction compared with other NMR signals, was assigned to the alcohol product resulting from the reduction of the hydrolysis product aldehyde of **P1** (Figure S11).

Overall, the well resolved NMR signals of free ^19^F-probe, its GSH adduct, and additional reductions of ^19^F-probe and its hydrolysates as demonstrated in Figure 4b enable one to quantify the GSH level in the live cells by ^19^F-NMR. This GSH quantification method is not biased by the reduction of the probe by the intracellular reductases, as the reduction fractions can be readily subtracted by the total concentration of ^19^F-probe based on the integrals of NMR peaks. According to the formula of the dissociation constant *K*_d_ = [GSH][**P**]/[**P**-SG], we generated a linear correlation between [**P**-SG]^−1^ and [**P1**]^−1^ (details in the Supporting Information). The determined overall *K*_d_ of **P1**-SG in the NMR tube was 0.15 ± 0.02 mM, and the content of GSH in each single HeLa cell was about 16.9 ± 2.3 fmol at 298 K. It is noted that the stable GSH adduct formed with the irreversible ^19^F-probe is readily excreted from the intracellular media, which will be hydrolyzed in the cancer cell membrane (Figure S12).^21^ In general, the advantage of this reversible formation of the ^19^F-probe with GSH over an irreversible manner prevents excretion or leak of intracellular GSH. Based on the above observations, we outlined a general conclusion on the in-cell detecting strategy by ^19^F-NMR (Figure 4e).

In order to examine the suitability of **P1** for the intracellular GSH quantification in different cell lines, the human normal embryonic kidney cell (HEK293T), mouse embryo fibroblast cell (NIH-3T3), human liver carcinoma cell (HepG2), and human lung adenocarcinoma cell (A549) were used for efficacy evaluation (Figures S13–S15). We first proceeded with the in-cell GSH assay at 298 K, and the concentration of intracellular GSH was calculated by measurement of the cell volume as determined (Table S4), and the determined GSH level per cell was shown in Table 1. The GSH level in cancer cells is generally higher than that in the normal cells, and the content of GSH in A549 cells is highest among the tested cell lines, whereas NIH-3T3 has the lowest intracellular GSH level. The results are consistent with the fact of metabolic changes in the cancer cells, which produce more ROS and require more antioxidants to maintain the redox balance.^3^ It is notable that the *K*_d_ values of the **P1**-SG adduct determined in different cell lines differ greatly (Table 1). These striking variations of the *K*_d_ values in different cells suggest a distinct impact of the intracellular environment on the formation of **P1** with the GSH adduct. Interestingly, *K*_d_ is generally larger in normal cells than in the cancer lines, in which the NIH-3T3 cell presents the largest *K*_d_ (0.52 mM), and the **P1**-SG adduct is most stable in the A549 cells (Table 1). For a better comparison, we have quantified the contents of GSH and *K*_d_ of **P1**-SG in the respective cell lysates (Table 1 and Figure S16). Because the stability of GSH between normal and cancer cells varies greatly,^21^ preparation of the cell lysates by normal lysis buffer or freezing-thawing process generally introduces hydrolysis of GSH in the cancer cells, which compromises the quantification of intracellular GSH. To minimize the GSH hydrolysis in cancer cells, we optimized the protocol of cell lysate preparation by immediate treatment of the collected cells in a boiling water bath to inhibit the enzymes related to the GSH metabolism. Overall, the *K*_d_ of the **P1**-SG adduct increases from the live cells to the respective cell lysates, suggesting the intracellular milieu is more favorable to form the **P1**-SG adduct. In general, the *K*_d_ value of **P1**-SG in cell lysates shows no significant differences between cancer and normal cells. It is notable that the GSH level between the live cell and cell lysates has distinct variations on the tested cancer and normal cells. As to the cancer cells, the intracellular GSH content is similar to that of cell lysates. In contrast, for the normal cells including HEK293T and NIH 3T3 cells, the determined GSH in cell lysates is generally 30% lower than that in the live cell (Table 1 and Figure S17). We also compared the GSH quantification in cell lysates by using the commercial kit and irreversible ^19^F probe,^21^ and similar results were obtained (Figure S18). However, these two methods are unable to quantify the GSH level in live cells.

**Table 1:** GSH Concentration in Different Cell Lines and the Respective Cell Lysates and the Concentration of GSH in Single Cell Was Determined by ^19^F-NMR and Respective Cell Volume

	in live cells			in cell lysates		
cell line	GSH (fmol/cell)	GSH (mM)	*K*_d_ (mM)	GSH (fmol/cell)	GSH (mM)	*K*_d_ (mM)
NIH-3T3	12.5 ± 1.3	3.5 ± 0.5	0.52 ± 0.01	8.2 ± 0.8	2.3 ± 0.3	0.38
HEK293T	14.3 ± 0.5	5.6 ± 1.1	0.29 ± 0.07	9.9 ± 0.5	3.9 ± 0.8	0.36
HeLa	16.9 ± 2.3	6.0 ± 0.8	0.15 ± 0.02	17.8 ± 0.8	6.3 ± 0.4	0.34
HepG2	19.0 ± 1.2	6.7 ± 0.6	0.17 ± 0.04	20.1 ± 0.2	7.0 ± 0.5	0.31
A549	35.9 ± 0.8	8.0 ± 0.7	0.13 ± 0.02	40.6 ± 1.0	9.0 ± 0.8	0.28

Many proteins contain free thiols, and it is believed that these proteins might contribute to intracellular homeostasis of biothiol pools.^39^ Peroxiredoxins are highly abundant in cells and contain a reactive cysteine to modulate the peroxide signaling,^40^ as these proteins are prone to form oligomers in solution and it is not known whether these proteins are reactive to the ^19^F-probe. Any formation of a protein thiol-probe adduct would produce the ^19^F-signals that contribute to either broad NMR peaks or biased baselines. However, no additional NMR signals in the live cell samples are determined. Because of GSH hydrolysis on the cell membrane of cancer cells,^21^ we used heat-treatment to prepare the cell lysates by deactivation of the enzymes involved in GSH hydrolysis (the details were in Supporting Information). The difference between the normal and cancer cells might be related to variations of intracellular GSH homeostasis upon heat treatment (Figure S17). During the preparation of cell lysates, it is plausible that the GSH is converted to thioether adducts^41^ or is oxidized by ROS into sulfenate, sulfinate or sulfonate,^42^ which cannot be reduced to free thiol by TCEP. In addition, one could not rule out the possibility of similar reactions of farnesylation or palmitoylation. The large variations in the measured GSH content between live cells and cell lysates in normal cells suggest either the unknown mechanism of GSH serving as an additional nucleophile in cell lysates or some proteins containing free thiols that are reactive to the free probe, which differs greatly from the cancer cells. Unfortunately, we do not know the exact explanation for this in the current stage.

**Figure 5:**
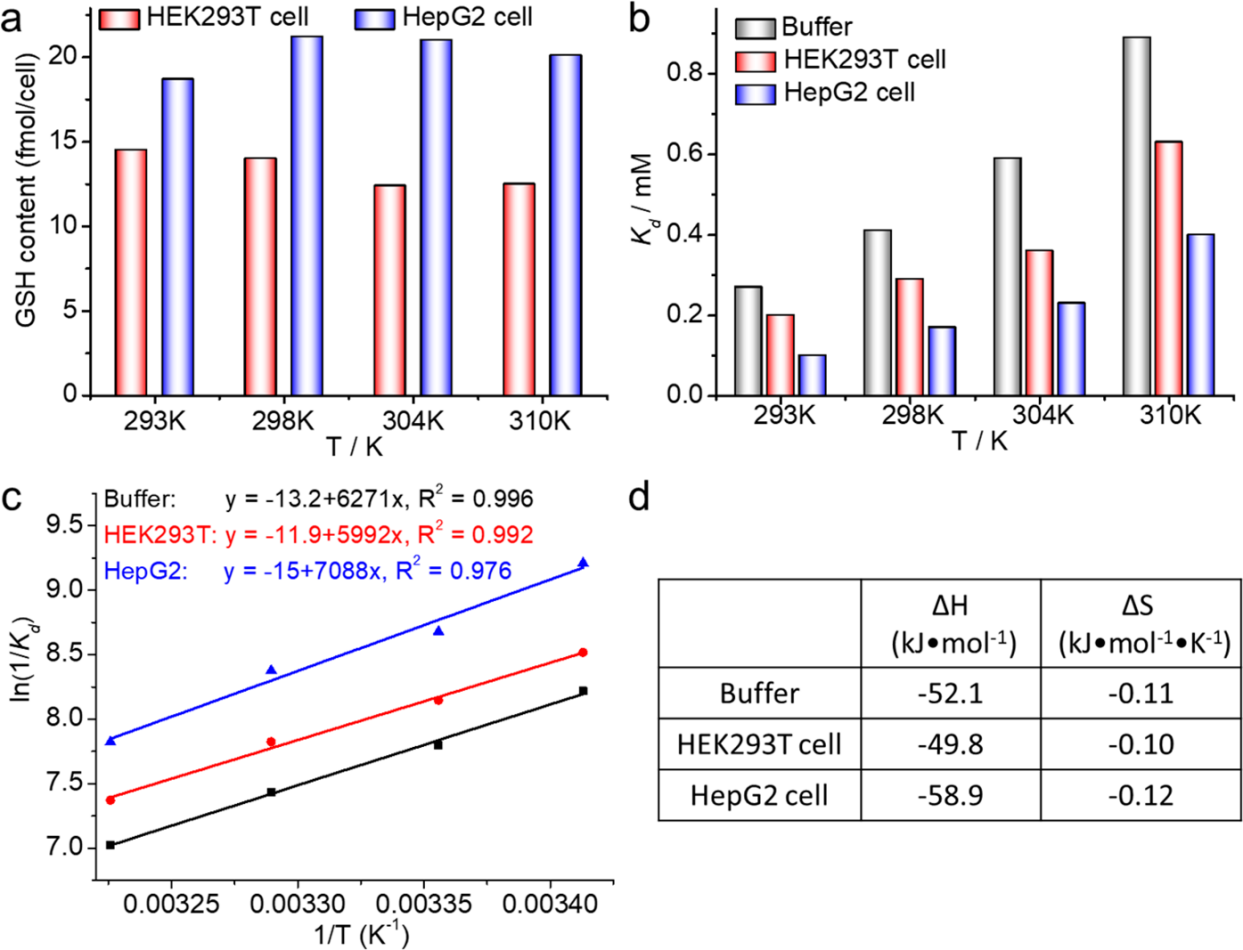
Quantification of intracellular GSH in live cells (HEK293T and HepG2) at 293, 298, 304, and 310 K, respectively, by using a reversible ^19^F probe. (a) The intracellular GSH level determined for HEK293T and HepG2 cells by ^19^F-NMR at different temperatures. (b) The dissociation constant *K*_d_, **P1**-SG, formed by **P1** and GSH in buffer and in live cells determined at different temperatures. (c) Linear correlation of ln(1/*K*_d_) and 1/*T* determined for **P1**-SG at different conditions. (d) Thermodynamic parameters determined in (c) for **P1**-SG in normal buffer and live cells. The *K*_d_ was determined similar to the protocol established in Figure 4.

We next elucidated the temperature effect on the intracellular GSH level and the stability of the **P1**-SG adduct. We first evaluated the reaction of **P1** with GSH in buffer conditions at 293, 304, and 310 K, respectively. Similar to Figure 2a, the reaction of **P1** with GSH produces identical products, and the respective *K*_d_ of **P1**-SG was determined accordingly following the same protocol as shown in Figure 4. We therefore assessed the feasibility of intracellular GSH quantification by using the **P1** probe at different temperatures, and two cell lines including HEK293T and HepG2 were used. Following the same protocol as that performed at 298 K, the intracellular *K*_d_ of **P1**-SG was first established by titration of **P1** into live cell samples. Accordingly, the respective *K*_d_ values at each temperature were determined. As shown in Figure 5, the *K*_d_ of **P1**-SG increases with temperature for the test cell lines, which is similar to the trend as the in vitro condition (Figure 5). However, the intracellular GSH level does not vary significantly in the tested temperature range. These data suggest that the intracellular GSH is tightly controlled in live cells, which is not sensitive to the variation of temperature from 20 to 37 °C. A linear plot of the ln(1/*K*_d_) with temperature (1/*T*) resulted in thermodynamic parameters including enthalpy and entropy for the reaction of **P1** with GSH both in vitro and live cells. The determined enthalpy and entropy values are −52.1 and −0.11 kJ/(mol·K) in buffer, −49.8 and −0.10 kJ/(mol·K) in live HEK293T cells, and −58.9 and −0.12 kJ/(mol·K) in live HepG2 cells, respectively (Figure 5d). It is notable that the reaction of **P1** with intracellular GSH is mainly driven by enthalpy instead of entropy, implying the distinct variations on the intracellular milieu between types of cell lines. We noted that the intracellular reduction rate of **P1** in live cells increases with temperature. The NMR signals of reduced **P1** are significantly higher at 310 K than at other temperatures, implying the higher reactivity of the enzymes involved in the reduction of the olefinic bond under physiological temperature (Figure S19).

### Real-Time Tracking the Variations of Intracellular GSH Level

**Figure 6:**
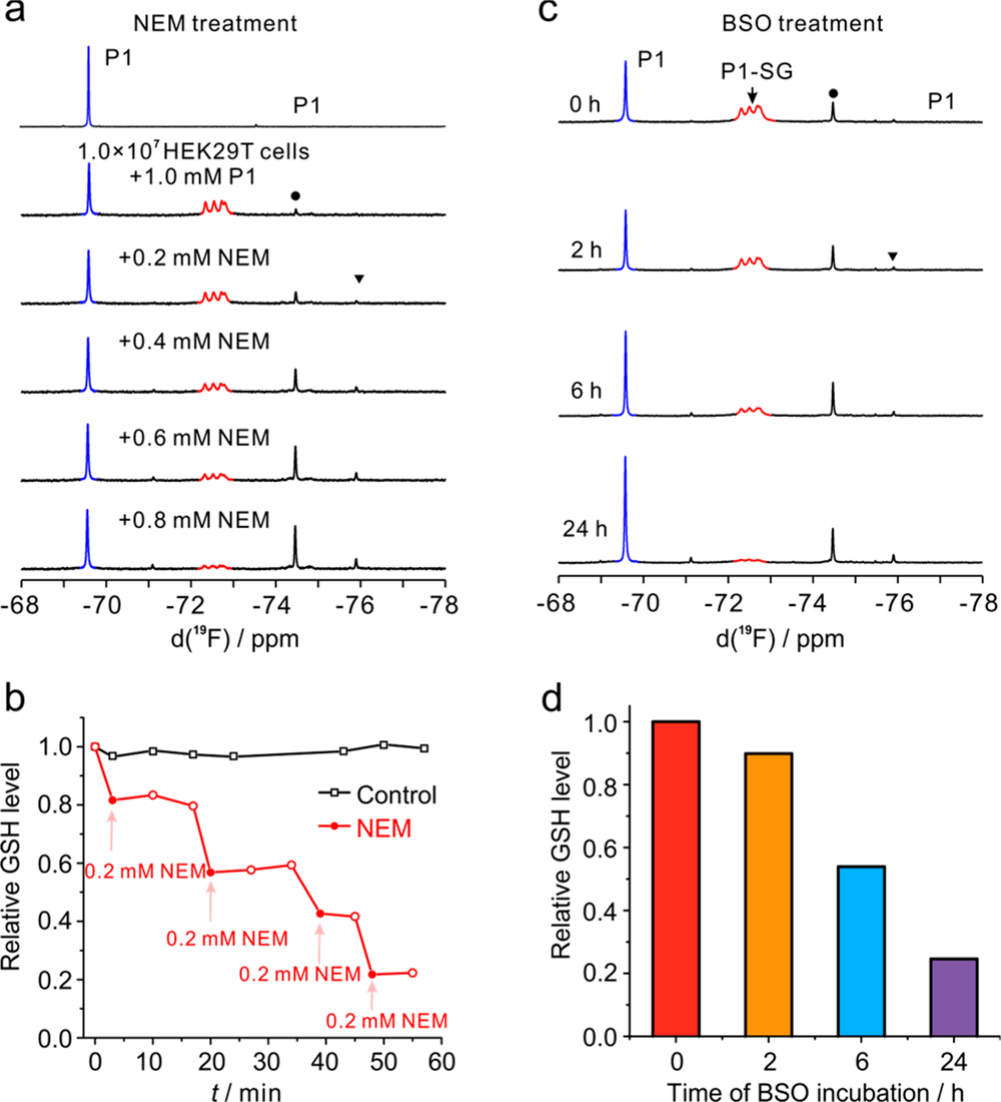
Real-time quantification of the intracellular GSH level in live cells upon treatment with NEM and BSO. (a) ^19^F-NMR spectra recorded for the sample of live HEK293T cells and **P1** with addition of NEM at each step, and each experiment was completed within a couple of minutes. (b) Relative GSH level upon addition of NEM as shown in (a), and the reference without addition of NEM was compared. The solid symbol denotes the point of addition of NEM and determined GSH level, and the open symbol denotes the time point measured for the GSH level. (c) ^19^F-NMR spectra recorded for mixture of 1 mM **P1** with the HepG2 cells after incubation with 0.5 mM BSO at 37 °C for 0, 2, 6, and 24 h, respectively. (d) GSH levels in HepG2 cells determined after incubation with BSO for varied time as shown in (c). All of the NMR experiments were recorded at 298 K.

The intracellular level of GSH correlates tightly with the steps of physiological processes including the homeostasis of the intracellular redox pool. We next quantified the intracellular GSH levels with respect to the different inducers. The intracellular GSH was first monitored by stepwise addition of thiol scavenger, NEM, and the 1D ^19^F NMR spectra were recorded. In general, one NMR titration experiment was completed within a couple of minutes. As shown in Figure 6a, the intracellular **P1**-SG adduct is uniformly disturbed in the live cell samples. The **P1**-SG product gradually decreases with the addition of NEM. The free **P1** does not increase linearly with the addition of NEM because certain fractions of free **P1** were reduced in the live cells. As mentioned above, the reduction of olefinic bond in **P1** was dominant over the hydrolysis product and does not interfere with the quantification of intracellular GSH by **P1**, which can be subtracted in calculations of the *K*_d_of **P1**-SG adduct. The intracellular GSH was quantified after each addition of NEM. It is evident that the irreversible reaction of NEM with GSH gradually exhausts the intracellular GSH with an increase of NEM; however, it remains stable in the absence of NEM (Figure 6b). It is noted that the addition of NEM does not introduce obvious hydrolysis of GSH because any modification or variation of the free thiol group will be encoded in the ^19^F-NMR spectrum.

l-Buthionine sulfoximine (BSO) is a specific inhibitor for GSH synthesis.^43^ It inhibits the activity of glutamate-cysteine synthetase (GCS) and increases the activity of GPX and the content of reactive oxygen species. BSO is reported to induce ferroptosis^44^ and can serve as a ferroptosis trigger.^45^ The HepG2 cells were incubated with 0.5 mM BSO for 0, 2, 6, and 24 h, respectively, and the respective cells were mixed with 1 mM **P1** for intracellular GSH quantification. The intracellular GSH level presents in a time-dependent manner with the BSO treatment (Figure 6c). The NMR signals of the **P1**-SG adduct vary significantly for the cells after incubation with BSO. In general, the intracellular GSH level decreases gradually with incubation time in the presence of BSO. The GSH level was depleted by about 80% for the cells after incubation with BSO for 24 h (Figure 6d). We noted that no additional small thiols including cysteine and H_2_S were produced by BSO treatment, as evidenced by the ^19^F-NMR, and it is conclusive that BSO only attenuates the intracellular free GSH level and has no obvious effect on the accumulation of cysteine or H_2_S. The cell viability treated with BSO at varied times was assessed by Trypan blue staining (Figure S20). The NMR assay for each sample fraction was completed within a couple of minutes. The cells were mostly alive, and no hydrolysis and export of GSH outside of the cells were determined. Overall, the data conclusively indicate that the reversible probe **P1** is able to quantify in real time the changes of intracellular GSH content at the initial state of the ferroptosis level by ^19^F-NMR.

## Conclusion

The abnormal variations of GSH are closely associated with pathological phenotypes, and quantification of GSH levels in live cells has witnessed significant progress in recent years. Real-time monitoring GSH levels offers insightful information on intracellular conditions; however, traceless and harmless detection methods are in high demand in this field. We herein showed that the ^19^F-probe reversibly forms adducts with biothiols under physiological conditions, but the chemical shifts of the adducts are distinguishable between Cys, GSH, and Hcy, which allows simultaneous quantification of these small biothiols if they are copresent in a biofluid. The readily formed GSH adduct has an appropriate *K*_d_ (0.41 mM) that enables the feasibility of quantification GSH in live cells by ^19^F-NMR. The live-cell NMR method indicates that the free ^19^F-probe readily crosses the cell membrane and is also exported outside of the live cells in a fast exchange regime, resulting in an averaged NMR signal. The ^19^F-probe forms an adduct with intracellular GSH reversibly in the cytoplasm, and the reversible association prevents free GSH and its probe adduct from leak or exportation from the intracellular media, which generally proceeds in the irreversible detection.^21^ This reversible detection enables high fidelity and accuracy in intracellular GSH quantification within a couple of minutes. The high performance of the ^19^F-probe in monitoring the intracellular GSH level by ^19^F-NMR has been demonstrated in different cell lines including NIH-3T3, HEK293T, HeLa, HepG2, and A549. The intracellular GSH level is readily quantified by the established ^19^F-NMR method, and remarkable stability variations of the GSH and probe adduct between normal and cancer cells have been determined. In addition, the reversible determination method allows one to monitor in real-time the GSH variations in the live cells upon inducer treatment.

Compared with isotope-labeled GSH or protein samples to monitor the GSH homeostasis in live cells,^31,32^ the reversible ^19^F-probe has the advantage of quantification of intracellular GSH level in real time without changing the cell growing media, which allows one to more closely monitor the GSH level with response to the variations of inducer or temperature. In addition, one NMR experiment can be generally completed within a couple of minutes for the 0.5 mM ^19^F-probe in the cell mixture, and the NMR time can be tunable according to the specific task. For long-time NMR measurements (over a few hours), it is advisable to use the established bioreactor systems to keep the cells in healthy condition, which are widely used for in-cell NMR experiments especially for the isotope-labeled proteins and nucleic acids.^24,25,46^ It is noted that structural information can be compromised due to the lack of sufficient isotope labeling by ^19^F-NMR, which has to be compared with isotope labeling of target molecules in some cases.

The ^19^F-NMR demonstrates an efficient way to quantify variations of intracellular GSH and its related cell activity. It provides an opportunity to characterize GSH-related activities in pathological phenotypes with atomic resolution and free of background signals. It would be even more insightful to distinguish the GSH levels and associated activity in individual organelles with atomic resolution, which is still to be resolved in the near future.

## Supporting Information Available

The experimental details containing the synthesis of the ^19^F-probes, stability assay, NMR analysis, cell growth and cell lysate preparation, figures and tables (PDF)
