# Supplementary material for: Real-Time Monitoring of the Level and Activity of Intracellular Glutathione in Live Cells at Atomic Resolution by 19F-NMR
Source: ACS Cent Sci. 2023 Jul 21;9(8):1623–32. doi: 10.1021/acscentsci.3c00385 (PMC10451033; doi:10.1021/acscentsci.3c00385)
Supplement: Supplementary file 1 — oc3c00385_si_001.pdf [file oc3c00385_si_001.pdf]

Supporting Information

Real-time Monitoring of the Level and Activity of Intracellular Glutathione in Live Cells at Atomic Resolution by ^{19}F-NMR

Chao-Yu Cui‡, Bin Li‡, Xun-Cheng Su*

* State Key Laboratory of Elemento-organic Chemistry, College of Chemistry, Nankai University, Tianjin 300071, China

* *Corresponding author:* Xun-Cheng Su*, E-mail:* xunchengsu@nankai.edu.cn

‡ These authors contributed equally to this work

Table of contents

1. Experimental Section 3

1.1 Synthesis of reversible ^{19}F probes **P1**-**P3** 3

1.2 Solution preparation 6

1.3 NMR analysis 6

1.4 Determination of the dissociation constant, K_d, formed by the ^{19}F-probe and GSH in vitro 7

1.5 Cell growth and in-cell sample preparation 8

1.6 Preparation of cell lysates 8

1.7 Cell viability assay of ^{19}F-probe impact on the mammalian cells 9

1.8 Measurement of the GSH concentration in live cells and cell lysates 9

1.9 Determination of enthalpy and entropy parameters of ^{19}F-probe in reaction with GSH in live cells 11

2.0 Real-time quantifying GSH level in live-cell 11

2.1 Assay of BSO effect on the intracellular GSH levels 11

2. Supporting Figures 12

3. Supporting Tables 30

4. References 32

1. Experimental Section

1.1 Synthesis of reversible ^{19}F probes P1-P3

Synthesis of (E)-2-cyano-3-(2-fluoropyridin-3-yl)acrylamide (P1)

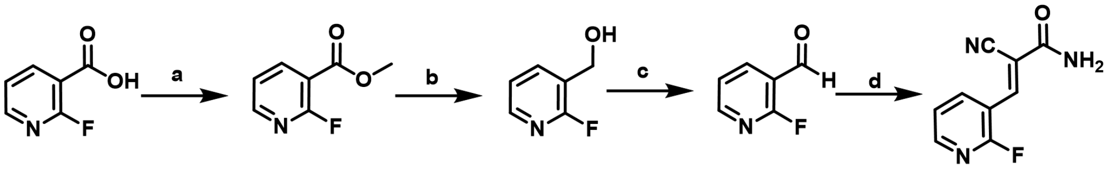

R-1 R-2 R-3 R-4 P1

Conditions: (a) 1) $(COCl)_2$, DMF, DCM, rt; 2) MeOH, 0°C; (b) $NaBH_4$, EtOH, reflux; (c) MnO_2, DCM, 40°C; (d) Cyanoacetamide, 1-Methylpiperazine, EtOH, rt.

Methyl 2-fluoronicotinate (R-2)

Oxalyl chloride (3.4 mL, 40 mmol) and N, N-dimethylformamide (15 µL, 0.2 mmol) was added to the solution of R-1 (2.8 g, 20 mmol) in 20 mL anhydrous dichloromethane. The reaction mixture was allowed to stir at room temperature for 4 h, and was then concentrated under reduced pressure. The residue was dissolved in 20 mL anhydrous methanol at 0°C, and was then stirred for 1 h. The above mixture was concentrated under reduced pressure and the title compound was purified by flash chromatography (2.6 g, 84% yield). ^{1}H NMR (400 MHz, $CDCl_3$) δ ppm: 8.45 – 8.37 (m, 2H), 7.35 – 7.30 (m, 1H), 3.97 (s, 3H). ^{13}C NMR (101 MHz, $CDCl_3$) δ ppm: 163.69 (d, J = 8.1 Hz), 161.53 (d, J = 249.8 Hz), 151.67 (d, J = 15.4 Hz), 143.25, 121.45 (d, J = 5.0 Hz), 113.72 (d, J = 25.0 Hz), 52.78. ^{19}F NMR (376 MHz, $CDCl_3$) δ ppm: -61.96.

(2-Fluoropyridin-3-yl)methanol (R-3)

Sodium borohydride (1.9 g, 50 mmol) was added to the solution of R-2 (1.55 g, 10 mmol) in 20 mL anhydrous ethanol at room temperature, and the mixture was heated to reflux for 2 h. The reaction was then quenched with 20 mL H_2O and the mixture was extracted with ethyl acetate. The combined organic layer was dried over anhydrous Na_2SO_4, and then concentrated. The residue was purified by flash chromatography to afford a white solid (0.86 g, 68% yield). ^{1}H NMR (400 MHz, $CDCl_3$) δ ppm: 7.98 (d, J = 4.2 Hz, 1H), 7.85 (t, J = 8.4 Hz, 1H), 7.12 (t, J = 5.4 Hz, 1H), 4.66 (s, 2H), 3.73 (s, 1H). ^{13}C NMR (101 MHz, $CDCl_3$) δ ppm: 160.77 (d, J = 239.7 Hz), 145.89 (d, J = 14.2 Hz), 139.56 (d, J = 5.6 Hz), 123.27 (d, J = 28.9 Hz), 121.69 (d, J = 4.3 Hz), 58.01. ^{19}F NMR (376

MHz, $CDCl_3$) δ ppm: -73.47.

2-Fluoronicotinaldehyde (R-4)

A mixture of R-3 (0.64 g, 5.0 mmol) and activated MnO_2 (4.38 g, 50 mmol) in 20 mL anhydrous dichloromethane was stirred at 45 °C for 12 h. The solid fraction was filtered off and washed with dichloromethane, and the filtrate was concentrated under reduced pressure to afford the title compound (0.3 g, 43% yield) as colorless liquid. ^{1}H NMR (400 MHz, $CDCl_3$) δ ppm: 10.29 (s, 1H), 8.46 (d, *J* = 3.6 Hz, 1H), 8.33 – 8.26 (m, 1H), 7.41 – 7.36 (m, 1H). ^{13}C NMR (101 MHz, $CDCl_3$) δ ppm: 186.30, 163.89 (d, J = 248.2 Hz), 153.30 (d, *J* = 16.0 Hz), 139.56 (d, *J* = 2.5 Hz), 122.32 (d, *J* = 4.5 Hz), 118.47 (d, *J* = 22.4 Hz). ^{19}F NMR (376 MHz, $CDCl_3$) δ ppm: -74.21.

(E)-2-cyano-3-(2-fluoropyridin-3-yl)acrylamide (P1)

A solution of R-4 (125 mg, 1.0 mmol), 2-cyanoacetamide (126 mg, 1.5 mmol) and 1-methylpiperazine (108 μL, 1.0 mmol) in 10 mL anhydrous ethyl alcohol was stirred at room temperature for 4 h. After the reaction was complete (monitored by thin-layer chromatography, TLC), the mixture was concentrated under reduced pressure and the left residue was purified by flash chromatography to afford a white solid compound (74 mg, 39% yield). ^{1}H NMR (400 MHz, DMSO-d_6) δ ppm: 8.57 (t, *J* = 8.2 Hz, 1H), 8.42 (d, *J* = 4.4 Hz, 1H), 8.19 (s, 1H), 8.12 (s, 1H), 7.92 (s, 1H), 7.62-7.55 (m, 1H). ^{13}C NMR (101 MHz, DMSO-d_6) δ ppm: 162.0, 160.5(d, J = 242.1 Hz), 151.3-151.0(m), 141.9 (d, *J* = 4.8 Hz), 140.7, 123.1, 115.8, 115.5, 112.3. ^{19}F NMR (376 MHz, DMSO-d_6) δ ppm: -67.74.

Synthesis of 2-cyano-3-(2-fluoropyridin-3-yl)acrylamide (P2)

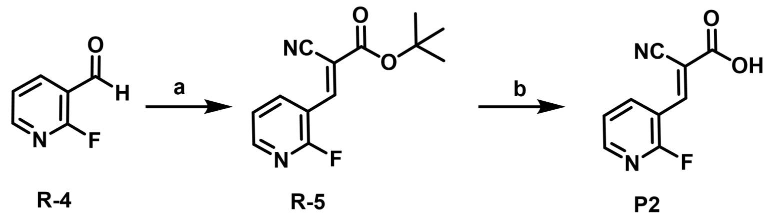

R-4 R-5 P2

Conditions: (a) tert-butyl 2-cyanoacetate, 1-methylpiperazine, EtOH, rt; (b) TFA, DCM, rt.

Tert-butyl 2-cyano-3-(2-fluoropyridin-3-yl)acrylate (R-5)

The mixture of R-4 (125 mg, 1.0 mmol), tert-butyl 2-cyanoacetate (211 mg, 1.5 mmol) and

1-methylpiperazine (108 µL, 1.0 mmol) in 10 mL anhydrous ethyl alcohol was stirred at room temperature for 5 h. After the reaction was complete (monitored by TLC), the reaction solution was concentrated under reduced pressure and the left residue was purified by flash chromatography to afford a white solid compound (175 mg, 70% yield). ^{1}H NMR (400 MHz, $CDCl_3$) δ ppm: 8.79 (t, *J* = 8.5 Hz, 1H), 8.39 (m, 2H), 7.45 – 7.35 (m, 1H), 1.61 (s, 9H). ^{13}C NMR (101 MHz, $CDCl_3$) δ ppm: 161.38 (d, *J* = 247.2 Hz), 151.59 (d, *J* = 15.5 Hz), 159.99, 143.96, 139.46, 122.21 (d, *J* = 4.5 Hz), 115.17, 114.91, 108.98, 84.65, 27.90.

2-Cyano-3-(2-fluoropyridin-3-yl)acrylic acid (P2)

5 mL trifluoroacetic acid was added to the solution of R-5 (248 mg, 1.0 mmol) in 5 mL anhydrous dichloromethane at room temperature. The above mixture was stirred at the same temperature for 2 h (monitored by TLC), and was then concentrated under reduced pressure. The left residue was purified by flash chromatography to afford a white solid compound (120 mg, 62% yield). ^{1}H NMR (400 MHz, DMSO-d_6) δ ppm: 8.68 – 8.58 (m, 1H), 8.45 (d, *J* = 4.0 Hz, 1H), 8.28 (s, 1H), 7.65 – 7.57 (m, 1H). ^{13}C NMR (101 MHz, DMSO-d_6) δ ppm: 162.74, 160.72 (d, *J* = 242.6 Hz), 151.84 (d, *J* = 15.1 Hz), 145.47 (d, *J* = 4.4 Hz), 140.73, 123.25, 115.49, 115.24, 109.41. ^{19}F NMR (376 MHz, DMSO-d_6) δ ppm: -67.61.

Synthesis of 2-((2-fluoropyridin-3-yl)methylene)malononitrile (P3)

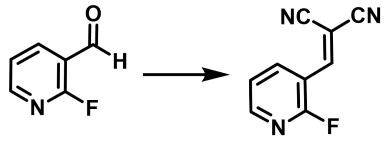

R-4 P3

Conditions: Malononitrile, 1-Methylpiperazine, EtOH, 70°C.

A mixture of R-4 (125 mg, 1.0 mmol), malononitrile (99 mg, 1.5 mmol) and 1-methylpiperazine (108 µL, 1.0 mmol) in 10 mL anhydrous ethyl alcohol was stirred at 70°C for 2 h. After the reaction was completed (monitored by TLC), the mixture was reduced under reduced pressure and the left residue was purified by flash chromatography to afford a yellow solid compound (74 mg, 43% yield). ^{1}H NMR (400 MHz, $CDCl_3$) δ ppm: 8.73 (ddd, *J* = 9.3, 4.9, 1.4 Hz, 1H), 8.50 (ddd, *J* = 4.8, 1.7, 1.0 Hz, 1H), 8.06 (s, 1H), 7.49 – 7.43 (m, 1H). ^{19}F NMR (376 MHz, $CDCl_3$) δ ppm: -65.94.

Synthesis of 2-cyano-3-(2-fluoropyridin-3-yl)propenamide (R-6)

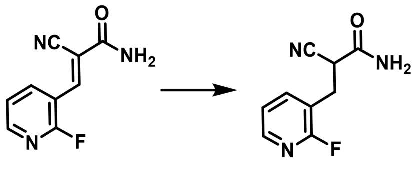

P1 R-6

Conditions: $NaBH_4$, MeOH, 0°C-rt.

To a solution of **P1** (95 mg, 0.5 mmol) in 5 mL anhydrous methanol was added sodium borohydride (95 mg, 2.5 mmol) at 0°C, and then the reaction mixture was left at room temperature for 0.5 h. The reaction was quenched with 5 mL H_2O and the reaction mixture was extracted with ethyl acetate. The combined organic extract was dried over anhydrous Na_2SO_4, and then concentrated. The residue was purified by flash chromatography to afford a white solid compound (70 mg, 73% yield). ^1H NMR (400 MHz, DMSO-d_6) δ ppm: 8.20 – 8.16 (m, 1H), 7.93 (ddd, J = 9.7, 7.4, 1.9 Hz, 1H), 7.86 (s, 1H), 7.57 (s, 1H), 7.37 (ddd, J = 7.0, 4.9, 1.9 Hz, 1H), 4.03 (dd, J = 8.3, 7.2 Hz, 1H), 3.24 – 3.11 (m, 2H). ^{13}C NMR (101 MHz, DMSO-d_6) δ ppm: 166.10, 161.80 (d, J = 236.4 Hz), 146.97 (d, J = 14.9 Hz), 143.02 (d, J = 5.1 Hz), 122.66 (d, J = 4.1 Hz), 119.04 (d, J = 30.8 Hz), 118.38, 37.88, 28.68. ^{19}F NMR (376 MHz, DMSO-d_6) δ ppm: -72.35.

1.2 Solution preparation

The probe **P1**-**P3** and N-ethylmaleimide (NEM) stock solution at 100 mM was prepared in dry N, N-dimethylformamide (DMF). 100 mM stock solution of GSH, amino acid, glucose, vitamin C was prepared in Milli-Q water. 30% Glycerol, 50% glycerol, 300 g/L ficoll400 and 100 g/L lysozyme were prepared in 20 mM phosphate buffer (PB, containing 6 mM KH_2PO_4 and 14 mM Na_2HPO_4, at pH 7.5). All in vitro NMR measurements were performed in 20 mM PB, at pH 7.5 and at 298K.

1.3 NMR analysis

NMR spectra were recorded at 293, 298, 304K and 310 K, respectively, on the Avance Neo 800 MHz NMR spectrometer equipped with a H(F)-TCI cryoprobe. All NMR data in vitro were collected with spectral width of 40 ppm centered at -72 ppm, 32k data points and 128 scans per experiment at 298K, and one experiment was generally completed within four minutes. The in-cell ^{19}F-NMR spectra were recorded with 32k data points with a spectral width of 40 ppm

centered at -72 ppm, and 512 or 1024 scans per experiment, which was completed within 13 or 26 minutes. To quantify the intracellular GSH, the dissociation constant K_d, formed by ^{19}F-probe and GSH, was first determined both in vitro and in live cells by titration experiment. In general, for the live cell samples, stepwise addition of P1 (concentration from 0.2, 0.4, 0.6, 0.8 to 1.0 mM) into the cell samples was performed and the respective NMR spectrum was recorded. The total experimental time was about 1.5 h (512 scans) or 3 h (1024 scans) for the five experiments. As to in vitro titration, titration of GSH into the solution of ^{19}F-probe was performed and similar experiment parameter was used for each experiment. Once the K_d was established for each cell line, the intracellular GSH level could be determined by recording 1D in-cell ^{19}F-NMR spectra with only addition of 0.2 mM **P1**, which takes less than 30 minutes. All NMR spectra were processed with Topspin 3.2 and analyzed with Topspin 2.1.

1.4 Determination of the dissociation constant, K_d, formed by the ^{19}F-probe and GSH adduct in vitro

To determine the K_d of the adduct formed by GSH and the ^{19}F-probe, [**P**-SG], we performed titration experiments of the probe, **P**, with the addition of different concentrations of GSH in 20 mM PB at pH 7.5. The reversible reaction between **P** and GSH can be written as in reaction (i) and equation (1).

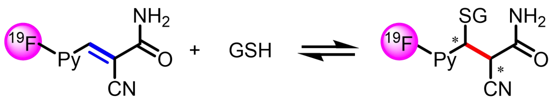

(i)

$$K_d=\frac{[\mathrm{P}][\mathrm{GSH}]}{[\mathrm{P\text{-}SG}]} \quad (1)$$

$$[\mathrm{P}] = [\mathrm{P}]_0 - [\mathrm{P}-\mathrm{SG}] \quad (2)$$

$$[\mathrm{GSH}] = [\mathrm{GSH}]_0 - [\mathrm{P}-\mathrm{SG}] \quad (3)$$

$$[\mathrm{P}-\mathrm{SG}] = \frac{[\mathrm{GSH}]_0 + [\mathrm{P}]_0 + K_\mathrm{d} - \sqrt{([\mathrm{GSH}]_0 + [\mathrm{P}]_0 + K_\mathrm{d})^2 - 4 \times [\mathrm{P}]_0 \times [\mathrm{GSH}]_0}}{2} \quad (4)$$

where [**P**], [GSH] and [**P**-SG] are the concentrations of free probe, GSH, and the adduct formed by the probe and GSH, respectively, in solution under the equilibrium condition. $[\mathrm{P}]_0$ and $[\mathrm{GSH}]_0$ are the initial concentration GSH and probe respectively. The $[\mathrm{P}]_0$ is fixed at 0.5 mM, while $[\mathrm{GSH}]_0$ is gradually increased from 0.2 mM to 4.0 mM. The corresponding concentrations of [***P***-SG] are

readily obtained by the integrals of fluorine signals. One can readily determine K_d by fitting the [P-SG] with $[GSH]_0$ following the Equation (4).

1.5 Cell growth and in-cell sample preparation

In general, mammalian cells (NIH-3T3, HEK293T, HeLa, HepG2 and A549) were seeded on 10 cm dishes and cultured in Dulbecco's Modified Eagle's medium (DMEM) with 10% fetal bovine serum (FBS) and 1% antibiotics (penicillin and streptomycin solution) at 37°C under 5% CO_2 humidified atmosphere. After incubation for 24 hours, the cells were washed twice with commercial PBS buffer (containing 10 mM Na_2HPO_4, 1.7 mM KH_2PO_4, 137 mM NaCl and 2.7 mM KCl) and then detached from dishes by treatment with 2 mL trypsin at 37°C for different times. The cells were resuspended in 4 mL DMEM containing 10% FBS to inactivate trypsin and were then gently centrifuged (200 × g) for 3 min. After being washed once with PBS, the cells were resuspended in DMEM with 10% D_2O (without FBS) and incubated with probe **P1** for 5 min. The suspension was transferred to a 3 mm NMR tube for subsequent in-cell sample analysis. After the in-cell NMR experiments, the cells were collected and the supernatant was checked for GSH leakage by NMR.

The cell volume was determined based on 1 mL cell suspension (~ 5×10^5 cells) by centrifugation at 2500×g for 1 min in the packed cell volume tubes (Sigma-Aldrich, cat. no. Z760986) following the protocol as previously reported.[1] Each measurement was repeated 3 times and averaged for each cell line.

1.6 Preparation of cell lysates

Method 1 (lysates-1): According to the above method, the cells were cultured until 95% confluency was reached. After being washed once with PBS, the cells ($4\sim10 \times 10^6$ cells) were resuspended in an Eppendorf tube containing 150 μL 20 mM PB buffer (pH 7.5). The Eppendorf tube was subsequently left in a boiling water bath for 5 min to lyse the cells. To ensure the cells are completely lysed, the resulting cell mixture was further lysed by multiple freeze-thaw cycles. The above sample mixture was frozen using liquid nitrogen and then thawed at 37°C water bath. After the cells were completely lysed, the mixture was centrifuged at 13000 rpm for 10 min to collect the supernatant (about 150 - 200 μL) and the cell debris (about 50-80 μL) was removed. The supernatant (about 150 μL) was mixed with 0.5 mM tris(2-carboxyethyl)phosphine (TCEP) for

subsequent NMR measurement.

Method 2 (lysates-2): The HEK293T or NIH-3T3 cells (~1.0×10^7 cells) were lysed with 150 μL lysis buffer (20 mM Tris-HCl pH 7.4, 150 mM NaCl, 1% NP-40, 5% glycerol, 2.5 mM sodium pyrophosphate, 1 mM β-glycerophosphate and 1 mM EDTA) at 0 °C for 15 min. Then, the supernatant (160~200 μL) was collected as described above for subsequent experiment.

1.7 Cell viability assay of ^{19}F-probe impact on the mammalian cells

Cell viability assay was performed by Trypan blue exclusion test. In general, HeLa cells (5×10^6) were cultured as described above and then collected in 150 μL DMEM (without FBS) followed by 1 mM **P1** incubation at 298K for 6 and 24 h, respectively. 20 μL cell suspension was mixed with 80 μL 0.4% Trypan blue. After 3 min, the cell viability was analyzed under a microscope.

1.8 Measurement of the GSH concentration in live cells and cell lysates

In general, the cells (NIH-3T3, HEK293T, HeLa, HepG2 ad A549) were cultured, and the fraction (containing 4～10 × 10^6 cells) was collected and resuspended in 150 μL DMEM containing 10% D_2O and then transferred into a 3 mm NMR tube. The live cell sample was titrated with increasing concentration of ^{19}F-probe from 0.2 to 1.0 mM, and the resulting mixture was monitored by 1D ^{19}F-NMR. The NMR signals were measured by recording 1D ^{19}F NMR spectra after each addition of the ^{19}F-probe. In contrast to the *in vitro* conditions, reduction of the ^{19}F-probe and its hydrolysis product proceeds in the live cells with time.

To quantify the intracellular GSH, the reversible reaction of ^{19}F-probe with GSH and irreversible reduction of the ^{19}F-probe by intracellular reductases have to be both considered, whereas the hydrolysis of ^{19}F-probe is negligible compared with the above two reactions. In addition to reaction (i), the reduction of the ^{19}F-probe in live cells has to be considered as shown in reaction (ii).

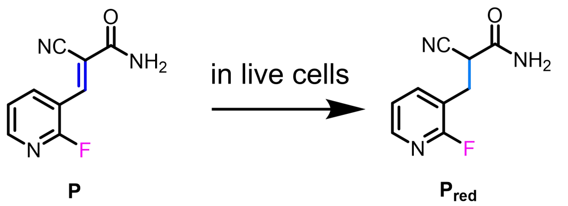

(ii)

The K_d of the adduct formed by ^{19}F-probe and intracellular GSH can be similarly determined as

shown in equation (1), in which the [**P**], [GSH] and [**P**-SG] denote the concentrations of free probe, free GSH, and the [**P**-SG] (the product formed by **P** and GSH in intracellular environment). As mentioned above, the concentration of intracellular [GSH] at equilibrium condition can be determined by the difference between the total concentration of GSH, $[GSH]_0$, and the concentration of the product formed by **P** and GSH, [**P**-SG]. Taken together, equation (1) can be rewritten as equations (5) and (6):

$$K_\mathrm{d}=\frac{[\mathrm{P}]([\mathrm{GSH}]_0\text{-}[\mathrm{P\text{-}SG}])}{[\mathrm{P\text{-}SG}]} \tag{5}$$

$$\frac{1}{[\mathrm{P\text{-}SG}]}=\frac{1}{[\mathrm{GSH}]_0}+K_\mathrm{d}\frac{1}{[\mathrm{GSH}]_0[\mathrm{P}]} \tag{6}$$

$$[\mathrm{P}]=\frac{\mathrm{I}_{[\mathrm{P}]}}{\mathrm{I}_{([\mathrm{P}]+[\mathrm{P\text{-}SG}]+[\mathrm{P_{red}}])}}\times[\mathrm{P}]_0 \tag{7}$$

$$[\mathrm{P\text{-}SG}]=\frac{\mathrm{I}_{[\mathrm{P\text{-}SG}]}}{\mathrm{I}_{([\mathrm{P}]+[\mathrm{P\text{-}SG}]+[\mathrm{P_{red}}])}}\times[\mathrm{P}]_0 \tag{8}$$

Under equilibrium conditions in live cells, the concentrations of free ^{19}F-probe, [**P**], and the adduct formed by probe and GSH, [**P**-SG], can be determined as shown in equations (7) and (8), respectively. $I_{[P]}$ is NMR peak area of the free probe, $I_{[P\text{-}SG]}$ is NMR peak area of the adduct formed by the probe and GSH, [P-SG]. $I_{([P]+[P\text{-}SG]+[Pred])}$ is the sum of NMR peak areas of free probe, the adduct formed by probe and GSH, and the reduced probe. $[P]_0$ is the total concentration of the probe. Therefore, the plot between 1/[P-SG] and 1/[P] in equation (6) results in the intercept and slop, and the reciprocal of the intercept gives the concentration of GSH and the slop divided by the intercept gives the K_d. Since the measured GSH concentration is the bulk concentration, intracellular GSH level was calculated according to the cell volume. The K_d and GSH measurements were repeated 3 times, and the value was averaged.

Similarly, quantification of GSH in cell lysates was determined and the fraction formed by TCEP and **P1** has to be considered, which can be readily identified in the 1D ^{19}F-NMR spectrum. In order to verify the reliability of the method, the total GSH level in cell lysates was further determined by irreversible probe, **^{19}F-NHS**, and commercial GSH and GSSG assay kit (Beyotime, China). The GSH quantification using irreversible probe **^{19}F-NHS** was performed as described in the previous report.[2] The quantification of GSH with commercial kit was performed according to

the Ellman's reagent, which is a thiol specific reaction reagent. The above methods used to determine GSH in cells lysates were all performed at 298K.

1.9 Determination of enthalpy and entropy parameters of ^{19}F-probe in reaction with GSH in live cells

The K_d value formed by probe **P1** and GSH adduct was measured in buffer and mammalian cells (HEK293T and HepG2 cell) at different temperatures. Assuming the enthalpy (ΔH) and entropy (ΔS) do not change with temperature, the enthalpy (ΔH) and entropy (ΔS) were calculated from van't Hoff equation[3]:

$$\ln\left(K^{\theta}\right) = \frac{\Delta \mathrm{S}}{\mathrm{R}} - \frac{\Delta \mathrm{H}}{\mathrm{RT}} \tag{9}$$

Here, T is the reaction temperature and R (8.314 J/(mol·K)) is the ideal gas constant. K^{θ} is equilibrium constant ($1/K_\mathrm{d}$). Equation (9) can be further derived as:

$$\ln\left(\frac{1}{K_\mathrm{d}}\right) = \frac{\Delta \mathrm{S}}{\mathrm{R}} - \frac{\Delta \mathrm{H}}{\mathrm{RT}} \tag{10}$$

With the value of K_d determined at 293, 298, 304 and 310 K, linear fitting of $\ln\left(\frac{1}{K_\mathrm{d}}\right)$ with respect to $\frac{1}{\mathrm{T}}$ results in the -ΔH/R as the slop and ΔS/R the intercept.

2.0 Real-time quantifying GSH level in live-cell

The cultured HEK293T cells were collected and resuspended in 150 µL DMEM containing 10% D_2O. ^{19}F-NMR spectra were recorded at different time after 1 mM **P1** treatment followed by various concentrations of NEM treatment. The ^{19}F NMR spectra were recorded at different times within 60 minutes at 298K using 256 scans, a recording time of 6 min per experiment. Similarly, the control experiment was performed in the absence of NEM. The NMR signal of **P1**-SG in live cells was monitored with incubation time.

2.1 Assay of BSO effect on the intracellular GSH levels

Fractions of the HepG2 cells were incubated with 0.5 mM BSO in cell culture medium (as described above) for different time (0 h, 2 h, 6 h and 24 h), and the cells were collected at different time courses. Each cell fraction (~4 × 10^6) was resuspended in 120 µL DMEM and then mixed with 1 mM **P1** for direct NMR measurements as described above. The ^{19}F NMR spectra were recorded at 298K using 256 scans, a recording time of about 6 min per each experiment.

2. Supporting Figures

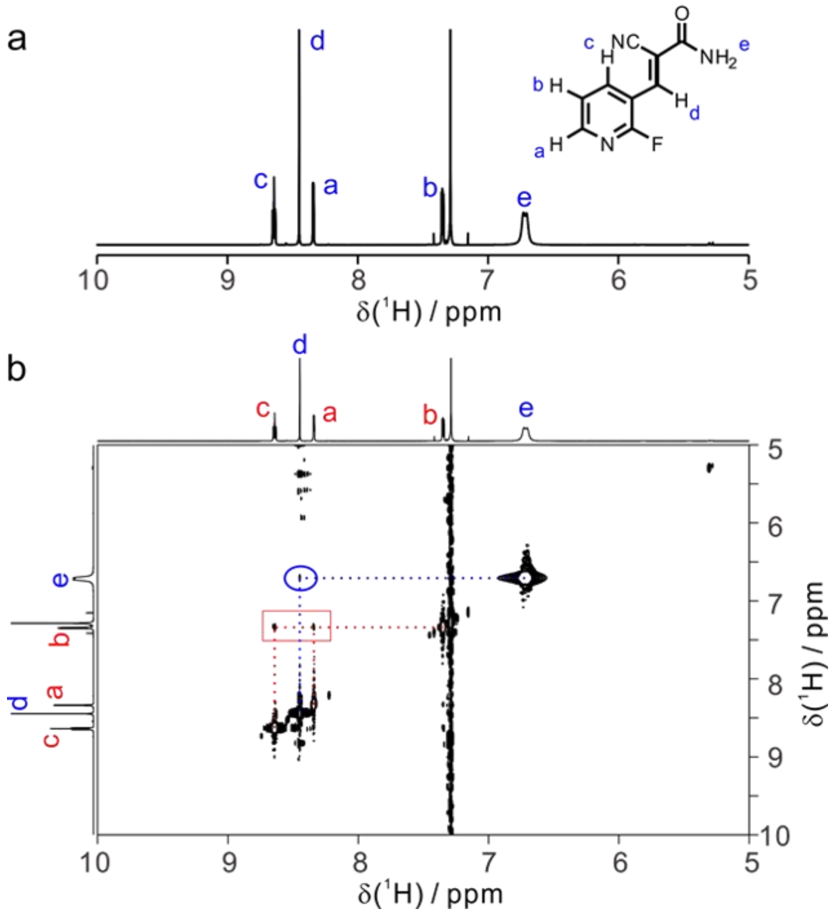

Figure S1. a) 1D ^{1}H NMR and b) 2D NOESY spectra recorded for **P1**. The spectra were recorded at 298 K with a ^{1}H NMR frequency of 800 MHz in $CDCl_3$.

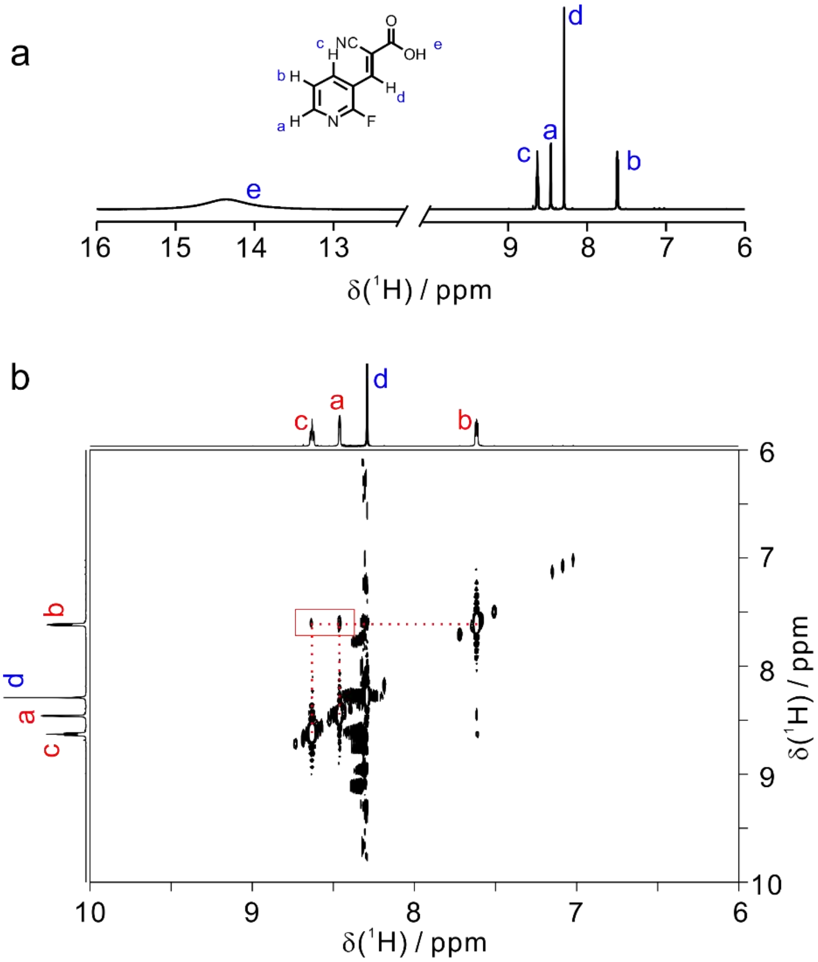

Figure S2. a) 1D ^{1}H NMR and b) 2D NOESY spectra recorded for **P2**. The spectra were recorded at 298 K with a ^{1}H NMR frequency of 800 MHz in DMSO-d6.

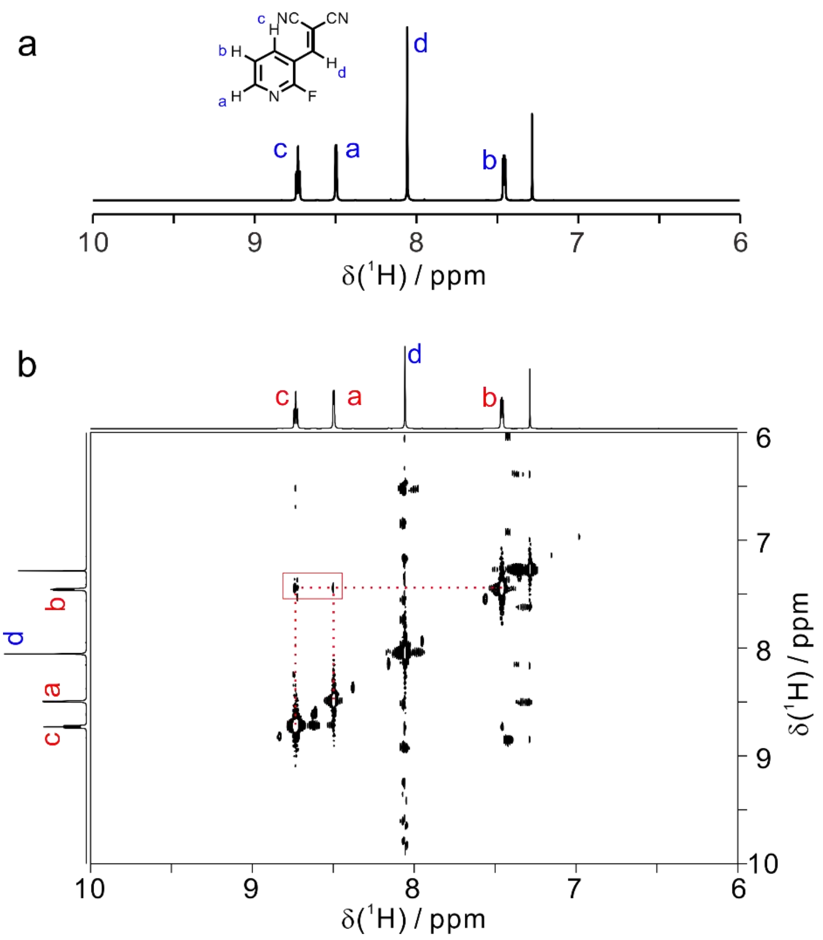

Figure S3. a) 1D ^{1}H NMR and b) 2D NOESY spectra recorded for **P3**. The spectra were recorded at 298 K with a ^{1}H NMR frequency of 800 MHz in $CDCl_3$.

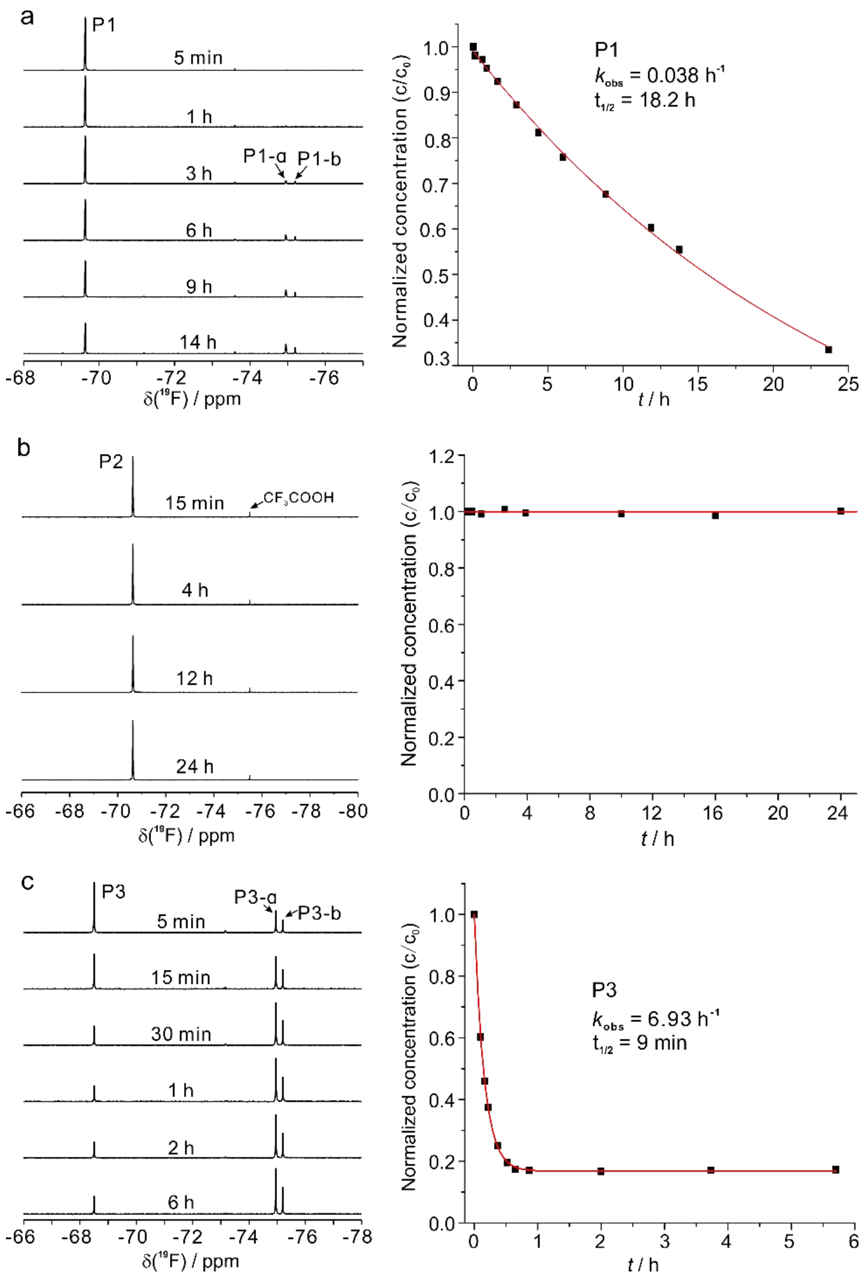

Figure S4. The stability assay of probe **P1-P3** in aqueous solution. Time-dependent 1D ^{19}F-NMR spectra recorded for the 0.5 mM ^{19}F-probe in aqueous solution with incubation time. a) **P1**, b) **P2**, c) **P3**. The overall hydrolysis reaction rate was determined by fitting the peak intensity with incubation time. The spectra were recorded at 298 K on a ^{1}H 800 MHz spectrometer in 20 mM phosphate buffer at pH 7.5.

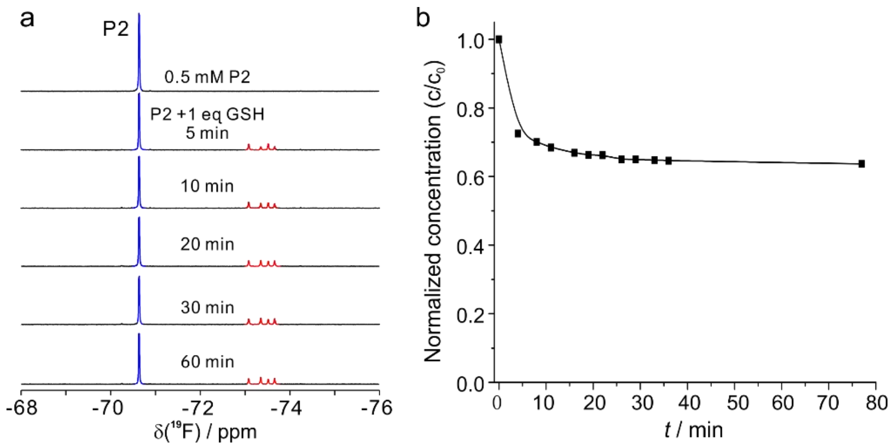

Figure S5. a) Time-dependent 1D ^{19}F-NMR spectra recorded for **P2** (0.5 mM) after addition of GSH (0.5 mM) in 20 mM phosphate buffer, pH 7.5. b) The plot of NMR peak intensity of free **P2** with time in solution. The spectra were recorded at 298 K on a ^{1}H 800 MHz spectrometer.

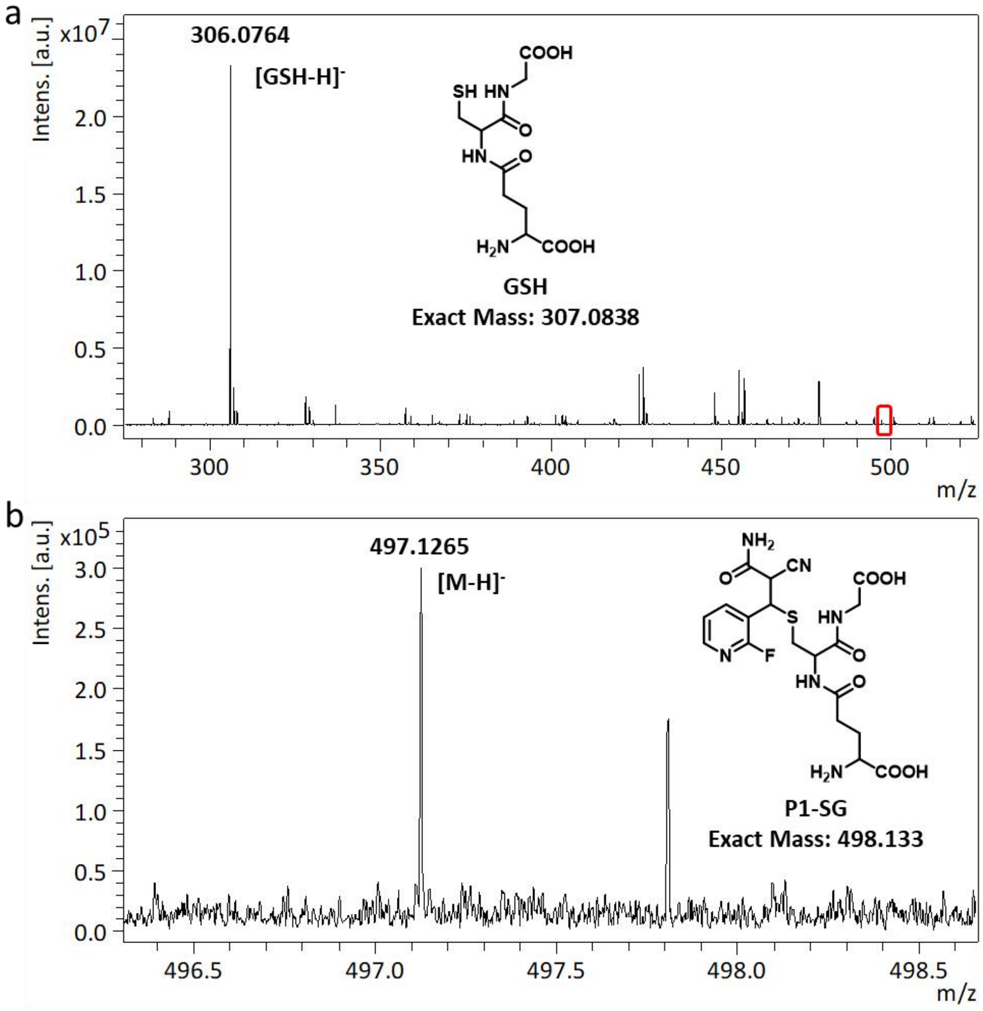

Figure S6. Mass spectra recorded for the diluted 50-fold of the reaction mixture of 0.5 mM **P1** and 2.0 mM GSH in 20 mM PB, pH 7.5. a) Full mass spectrum, b) enlarged view of the red box in a). The molecular mass and the chemical structure were labeled in the insertion. High resolution mass spectra were performed on a QFT-ESI mass spectrometer.

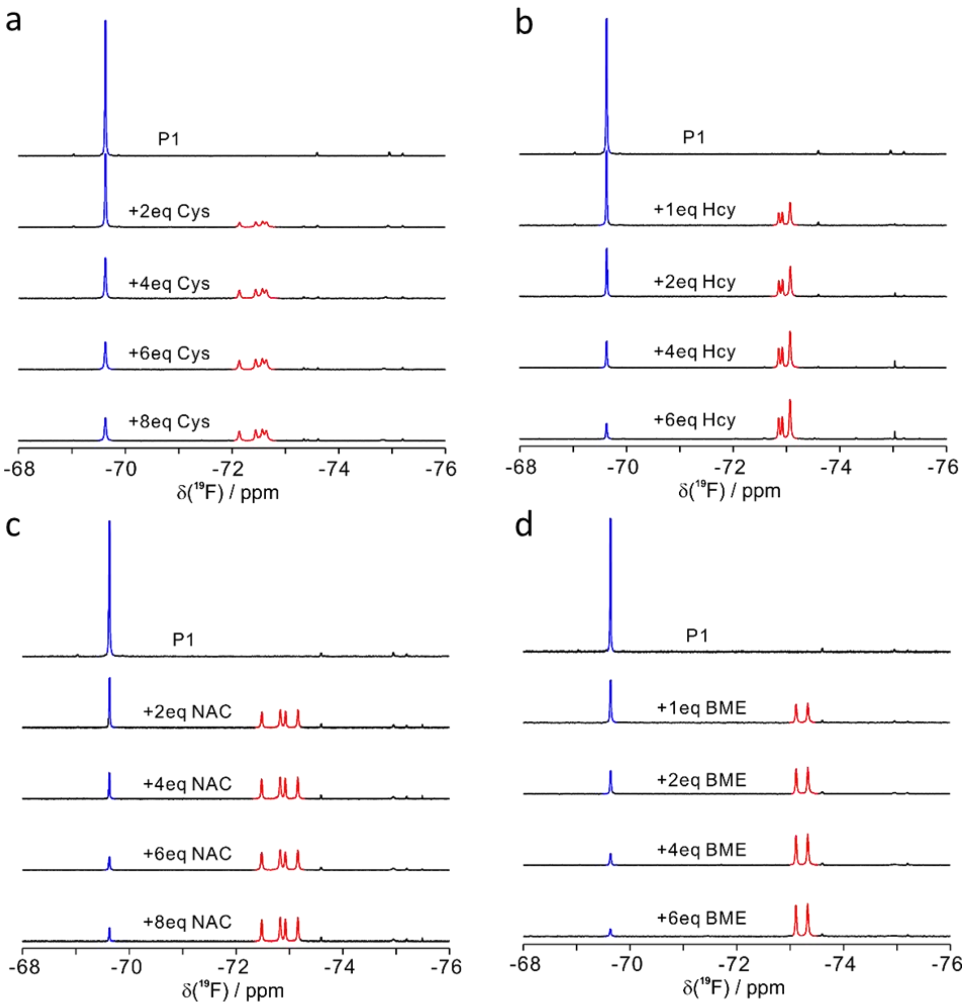

Figure S7. 1D ^{19}F-NMR spectra recorded for the mixture of 0.5 mM **P1** after addition of different concentrations of small thiol molecules. a) Cys, b) Hcy, c) NAC, d) β-mercaptoethanol (BME). The NMR signals of free **P1** and its thiol adducts were highlighted in blue and red, respectively. The NMR spectra were recorded on a ^{1}H 800 MHz spectrometer in 20 mM phosphate buffer at pH 7.5, and at 298 K.

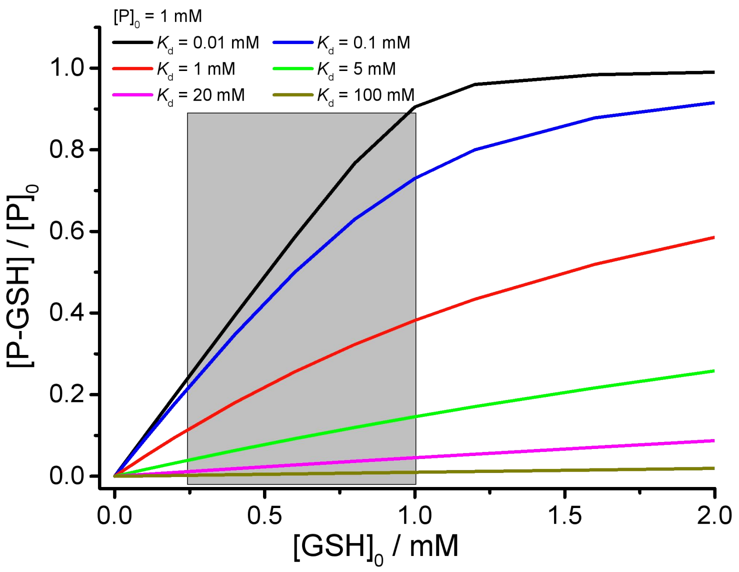

Figure S8. Correlation between the concentration of $[GSH]_0$ and the ratio of [**P**-SG]/$[\mathbf{P}]_0$ in the detecting solution with respect to the varied K_d, in which $[GSH]_0$ is the total concentration of GSH, [**P**-SG] and $[\mathbf{P}]_0$ are the concentration of **P**-SG adduct and total concentration of probe, respectively. For NMR detection, the fractions of [**P**-SG] and [**P**] have to be in decent levels to have a better signal-to-noise. The total concentration of GSH for suitable NMR detecting solution is highlighted in grey.

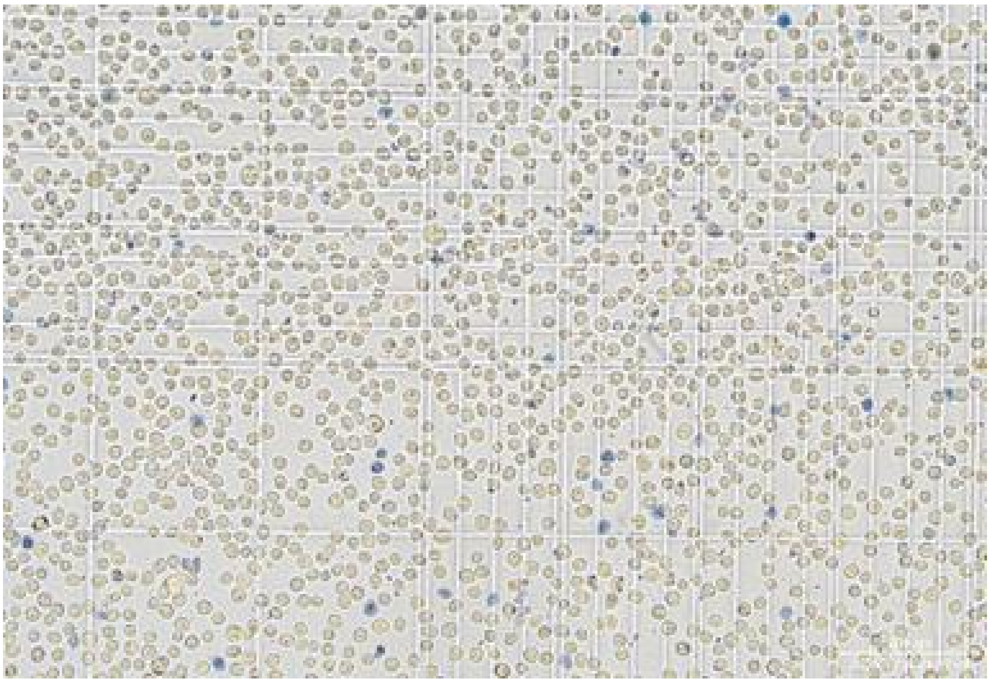

96% live cells (6 h)

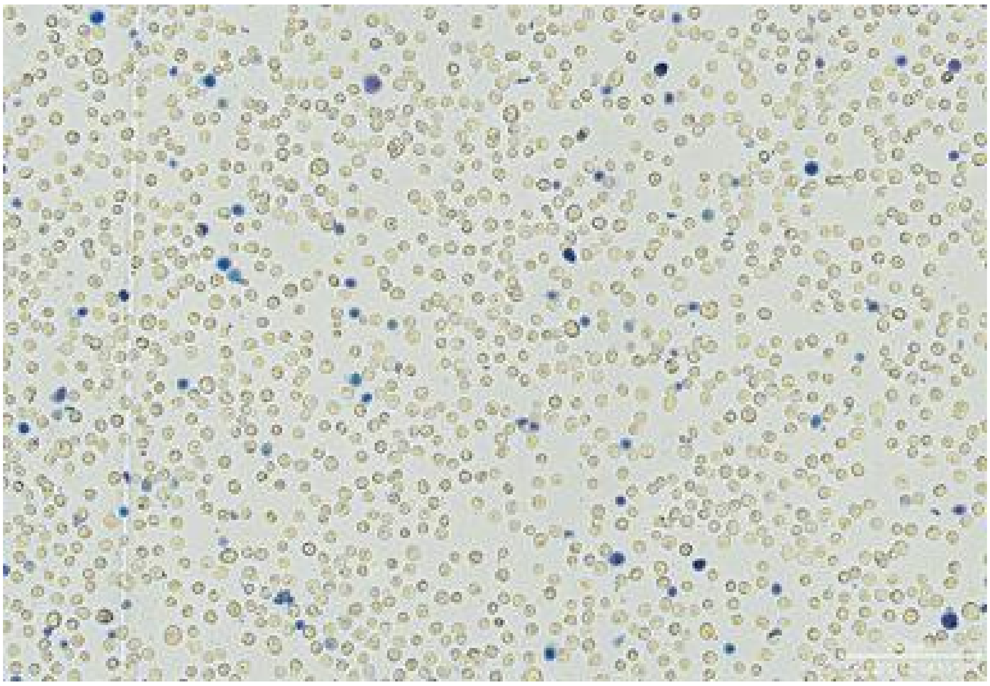

93% live cells (24 h)

Figure S9. Exemplary Trypan blue cell-viability test on HeLa cells after incubation with 1 mM **P1** at 298K for 6 h and 24 h, respectively.

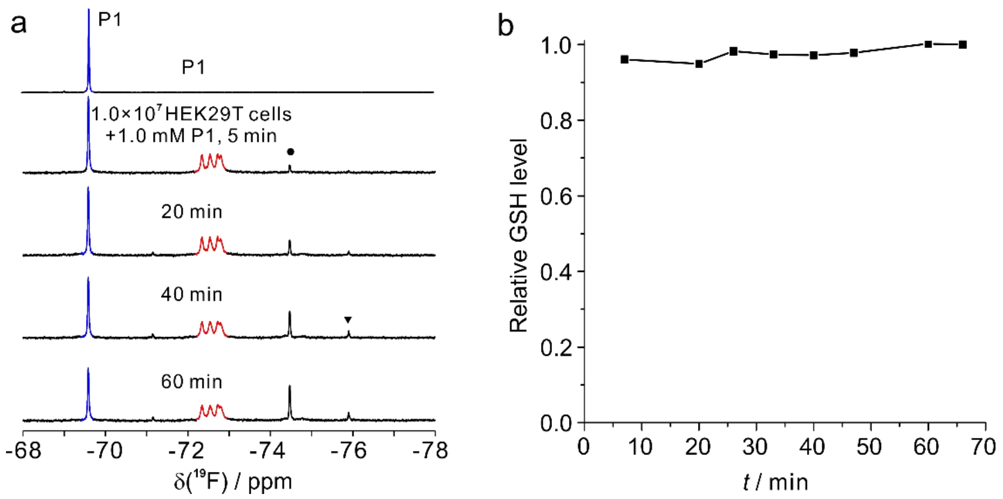

Figure S10. Real-time quantification intracellular GSH level. The HEK293T cells were mixed with 1.0 mM **P1**, and the reaction mixture was monitored by 1D ^{19}F-NMR. a) 1D ^{19}F NMR spectra recorded for the live sample of HEK293T cells after addition 1.0 mM **P1** for different time. For better comparison, the NMR spectrum of free **P1** in NMR buffer was shown in the top panel. b) Quantification of the real-time GSH content as shown in a). The spectra were recorded at 298 K on a ^{1}H 800 MHz spectrometer.

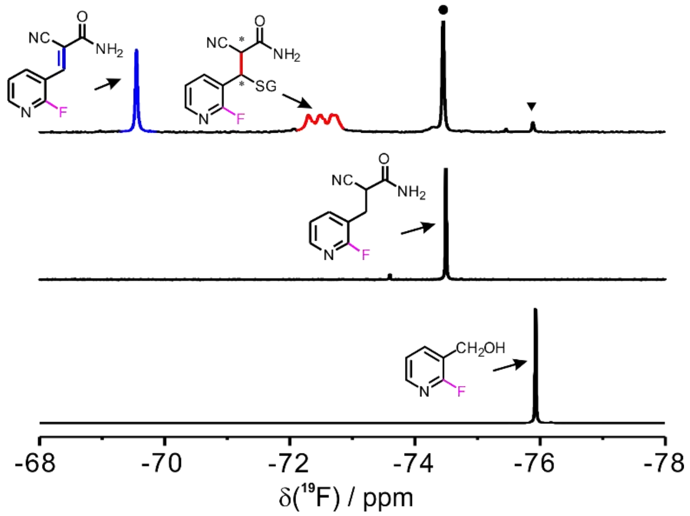

Figure S11. Assignment of the products observed in the mixture of **P1** after incubation with HeLa cells by comparison with the 1D ^{19}F-NMR spectra of standard compounds. It is evident that the additional peaks in the live cell samples (top) stem from the reduction of free **P1** (middle) and reduction of **P1** hydrolysis product (bottom), respectively.

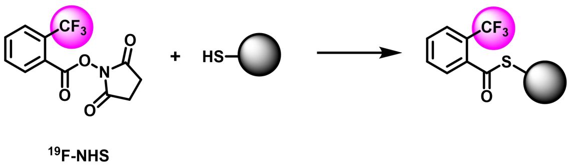

Scheme of quantification of biothiols with irreversible ^{19}F-probe

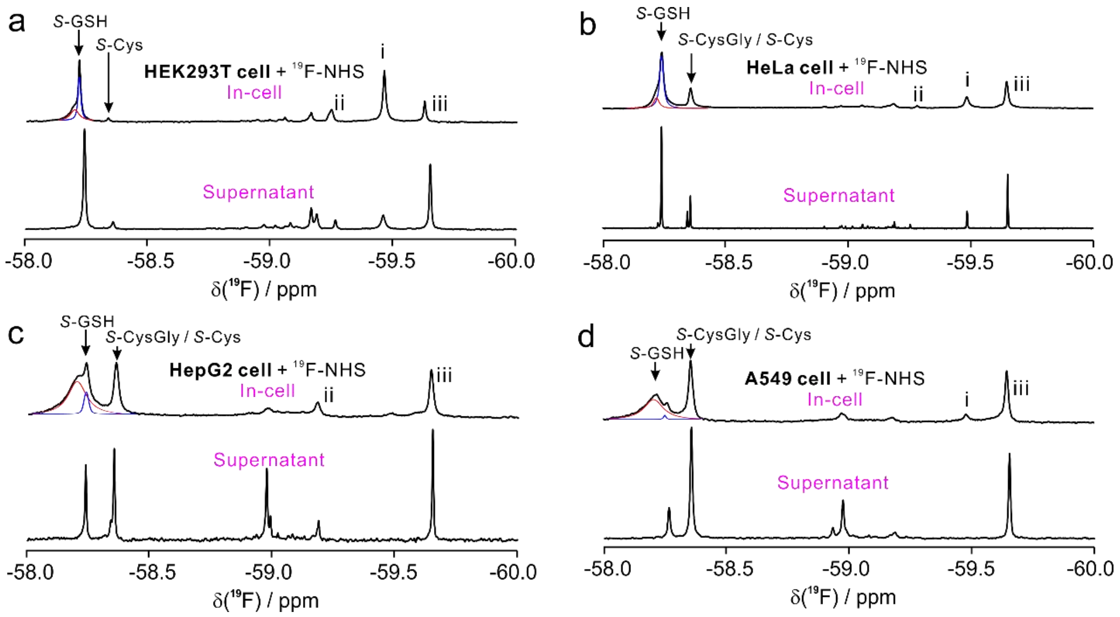

Figure S12. Scheme of quantification of biothiols with irreversible ^{19}F-probe. 1D ^{19}F-NMR spectra recorded for the mixture of **^{19}F-NHS** (1.0 mM) and live cells with incubation time, and selection of NMR spectrum at different time (top) and the supernatant after in-cell NMR experiments (down). a) NMR spectra of HEK293T cells after incubation with **^{19}F-NHS** for 5 h; b) NMR spectra of HeLa cells after incubation with **^{19}F-NHS** for 2 h; c) NMR spectra of HepG2 cell after incubation with **^{19}F-NHS** for 5 h; d) NMR spectra of A549 cell after incubation with **^{19}F-NHS** for 5 h. The raw data are black, and the deconvoluted peaks are highlighted in red and blue that represent the signals of intracellular and extracellular ***S*-GSH**, respectively. The signals of GSH and GSH metabolites are readily determined in the supernatant. The species labeled as i, ii and iii are the free **^{19}F-NHS**, hydrolysis intermediate and the hydrolyzed carboxylate, respectively, according the previous report.[2]

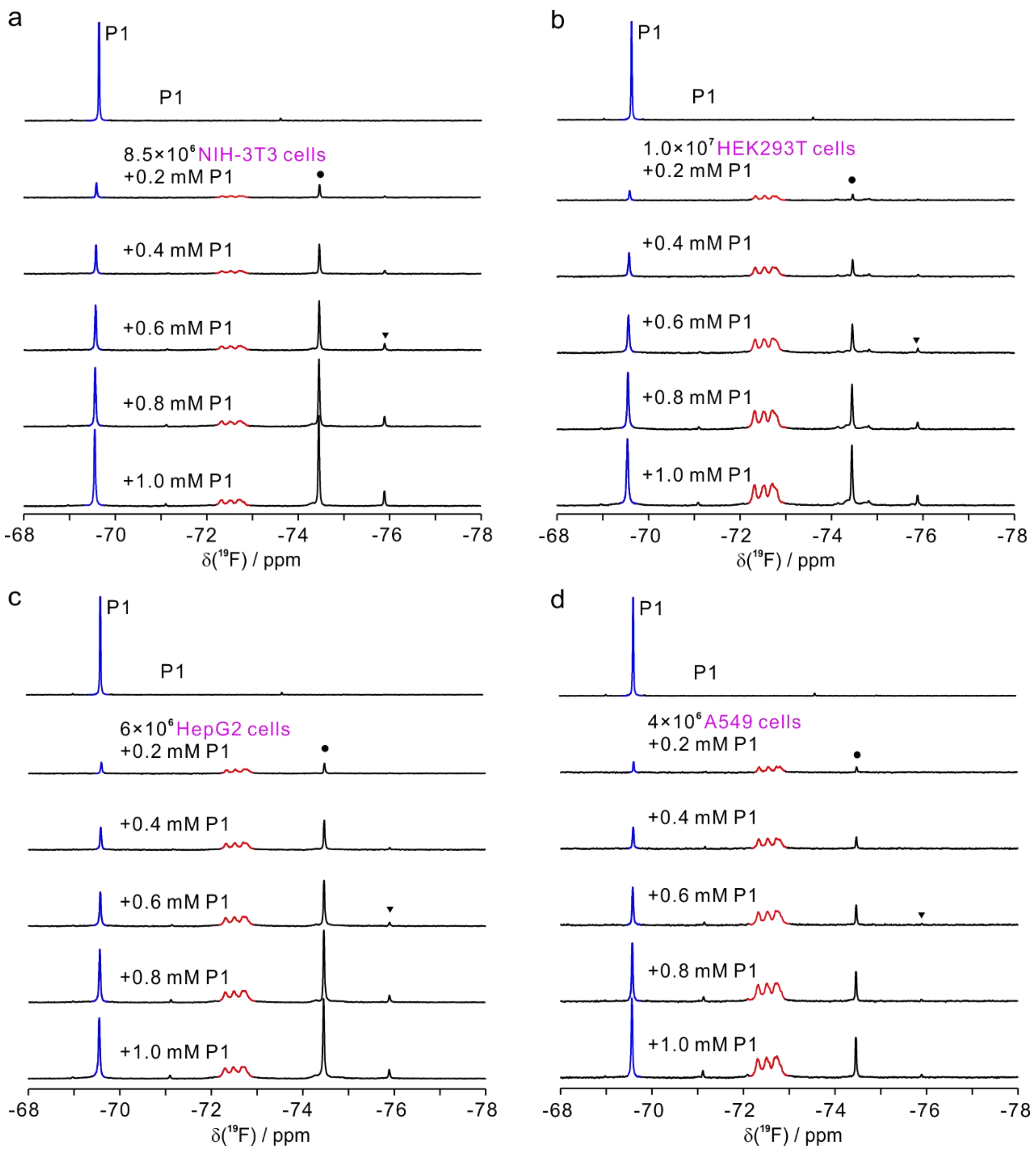

Figure S13. 1D ^{19}F-NMR spectra recorded for the live cells after addition of different concentrations of **P1**. a) NIH-3T3 cells, b) HEK293T cells, c) HepG2 cells, d) A549 cells. For better comparison, the NMR spectrum of free **P1** recorded in vitro condition was shown at the top panel for each cell line. The NMR signals of free **P1**, the adduct of **P1** with GSH, **P1**-SG, are highlighted in blue and red, respectively. The signal for the reduction product of free **P1** was labeled with solid circle and the reduction of aldehyde from the hydrolysis of **P1** was labeled with triangle. The NMR spectra were recorded at 298 K.

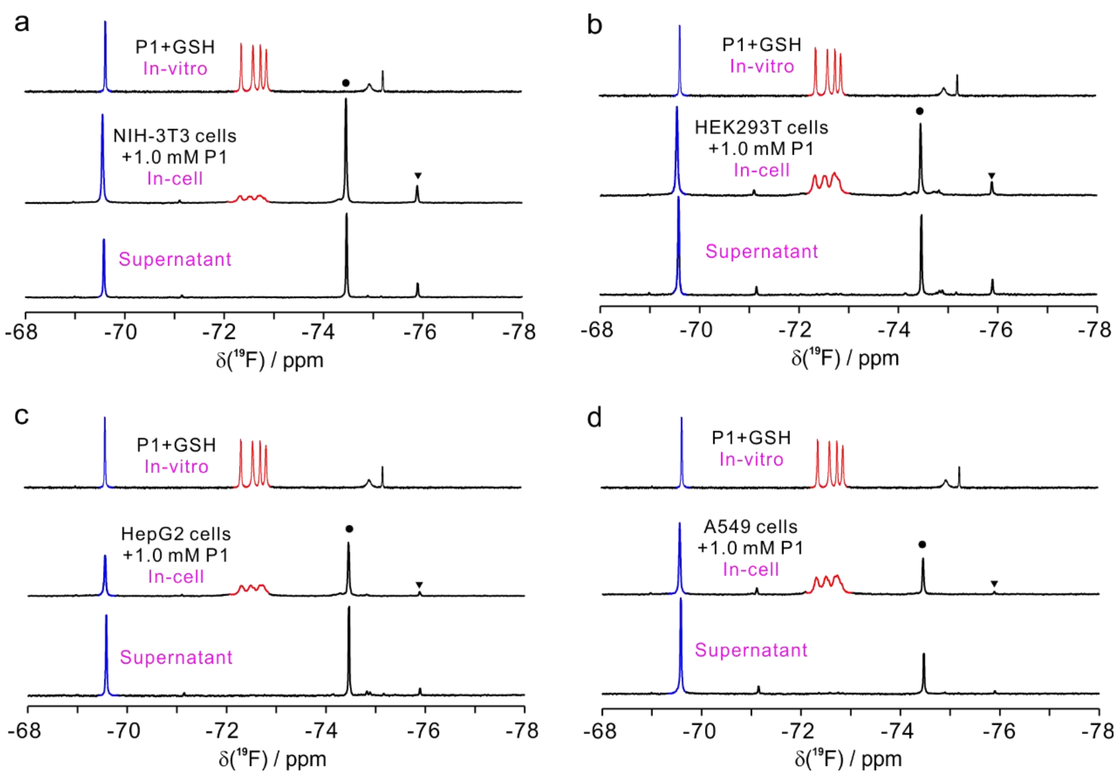

Figure S14. Comparison of 1D ^{19}F-NMR spectra recorded for **P1** (0.5 mM) with GSH (2 mM) in vitro (top), **P1** with live cells (middle), and supernatant of the **P1** with live cell sample after in-cell NMR measurement (bottom). a) NIH-3T3 cells, b) HEK293T cells, c) HepG2 cells, d) A549 cells. The NMR peaks of free **P1**, and the adduct of **P1** with GSH, are highlighted in blue and red, respectively. The signal for the reduction product of free **P1** was labeled with solid circle and the reduction of aldehyde from the hydrolysis of **P1** was labeled with triangle. It is evident that no GSH is leaked into the extracellular media during the in-cell measurement by the reversible quantification manner. The NMR spectra were recorded at 298 K.

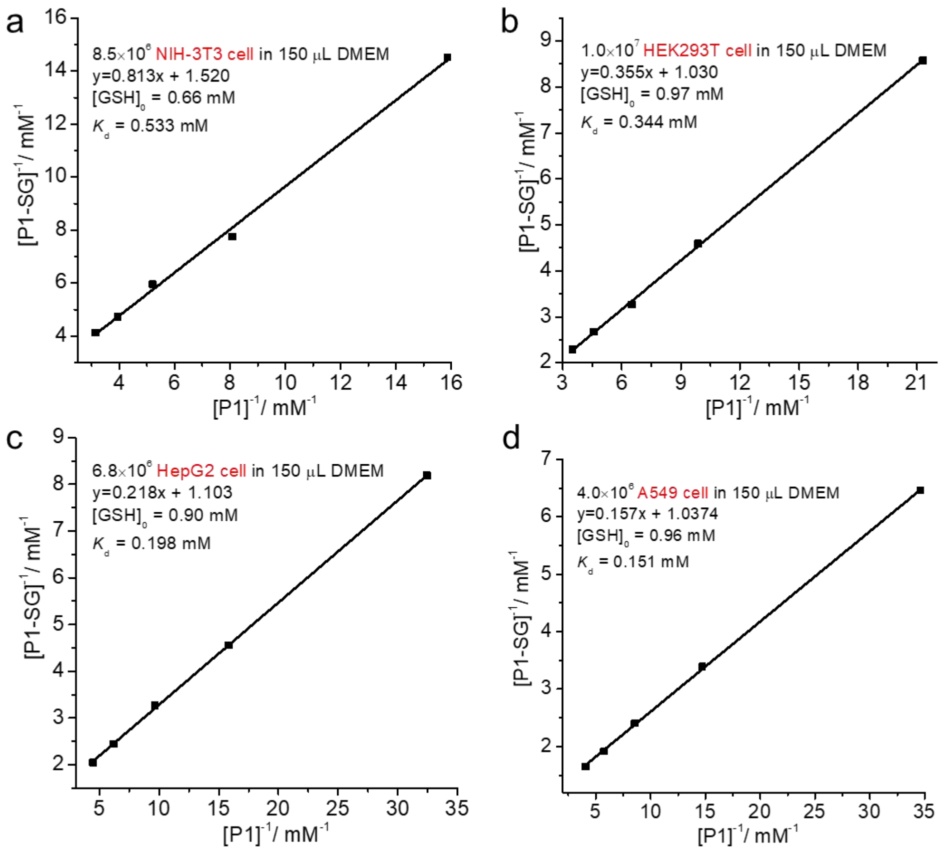

Figure S15. Linear correlation between the [**P1**-SG]$^{-1}$ and [**P1**]$^{-1}$ in the live cell samples in the 1D ^{19}F-NMR spectra for determination of K_d and the intracellular GSH level. [**P1**-SG] and [**P1**] are the concentrations in the live cell samples under equilibrium, and K_d is the dissociation constant of the **P1**-SG adduct. a) NIH-3T3 cell, b) HEK293T cell, c) HepG2 cell, d) A549 cell.

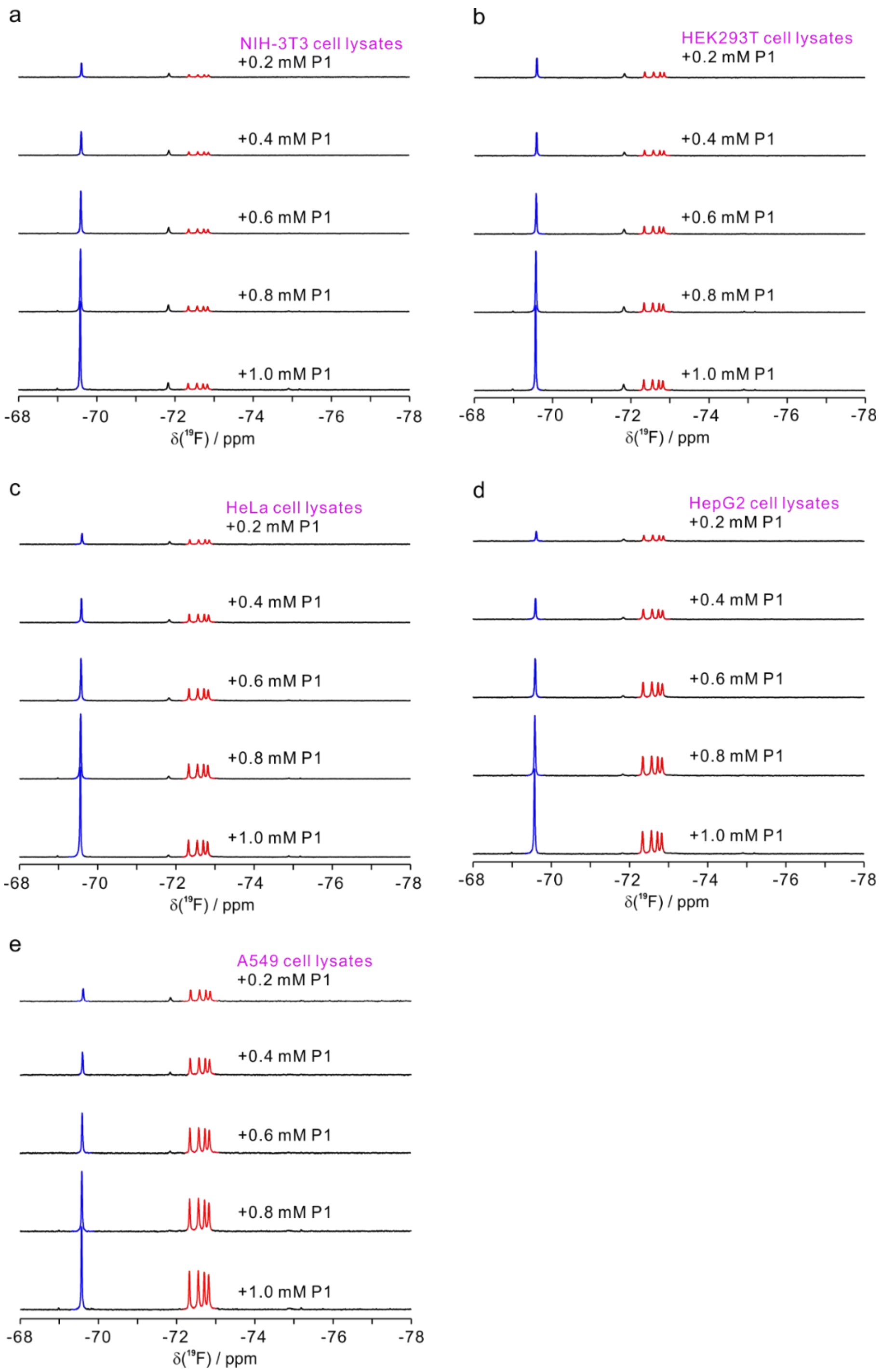

Figure S16. 1D ^{19}F-NMR spectra recorded for the sample of cell lysates with addition of **P1** for GSH quantification. a) NIH-3T3 cell lysates, b) HEK293T cell lysates, c) HeLa cell lysates, d) HepG2 cell lysates, e) A549 cell lysates. The NMR peaks of free **P1**, and the adduct of **P1** with GSH, [**P1**-SG], are highlighted in blue and red, respectively. The NMR signal at ~-71.8 ppm is of the product formed by **P1** and TCEP, which has to be considered for GSH quantification. The NMR spectra were recorded at 298 K.

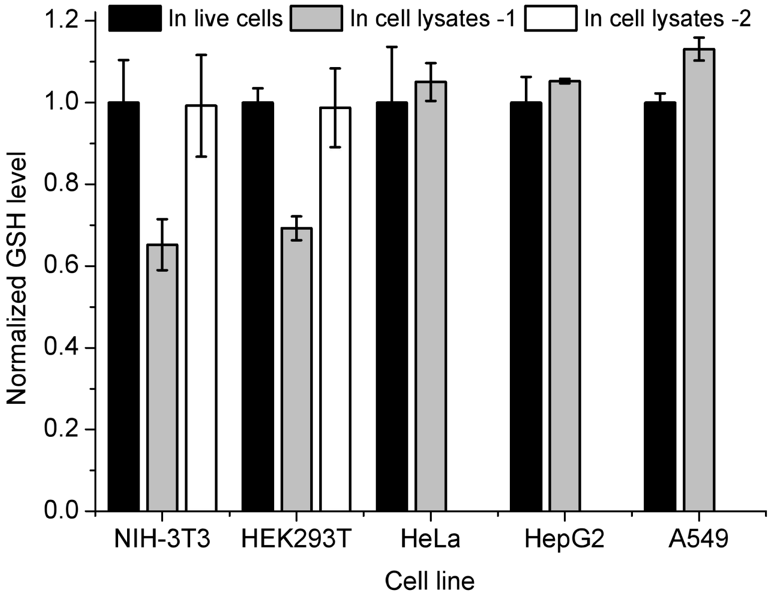

Figure S17. Comparison of GSH levels measured in live cells and lysates by reversible ^{19}F-NMR method for difference cell lines. Lysates-1 was prepared by method 1, and Lysates-2 was prepared by method 2.

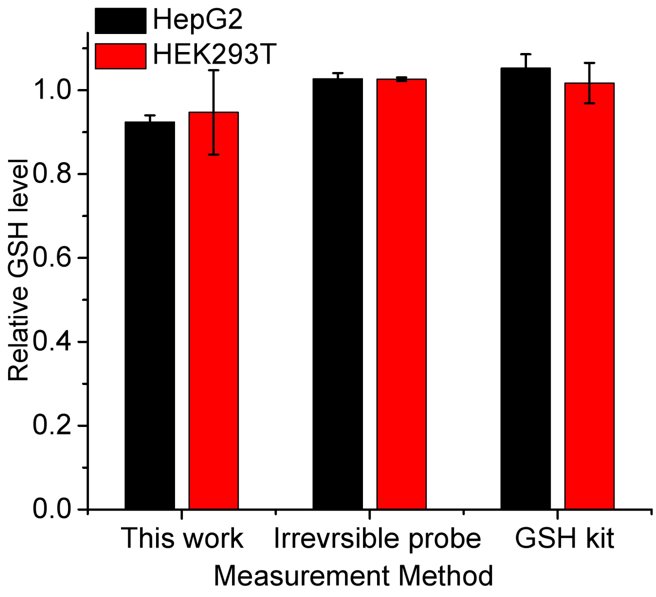

Figure S18. Quantification of GSH levels in HEK293T and HepG2 cell lysates (prepared with method 1, lysates-1) using reversible probe (**P1**), irreversible probe (**^{19}F-NHS**) and commercial GSH kit, respectively.

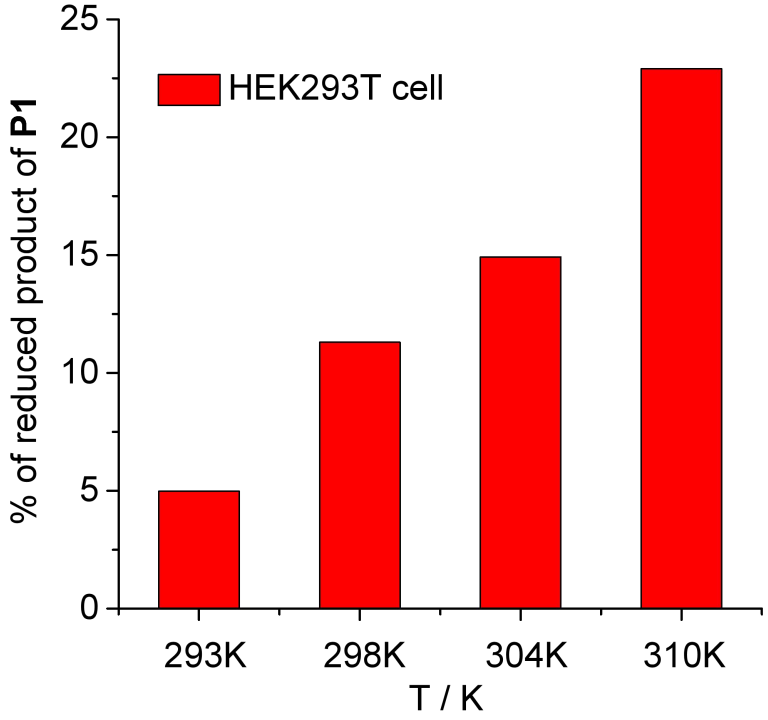

Figure S19. Reduction assay of **P1** in live cells with respect to temperature variations. The fraction of reduced **P1** in HEK293T cells at different temperatures was quantified, and the reduced product content was calculated by in-cell ^{19}F NMR spectra after mixing the live cells with 0.2 mM **P1** for 26 min.

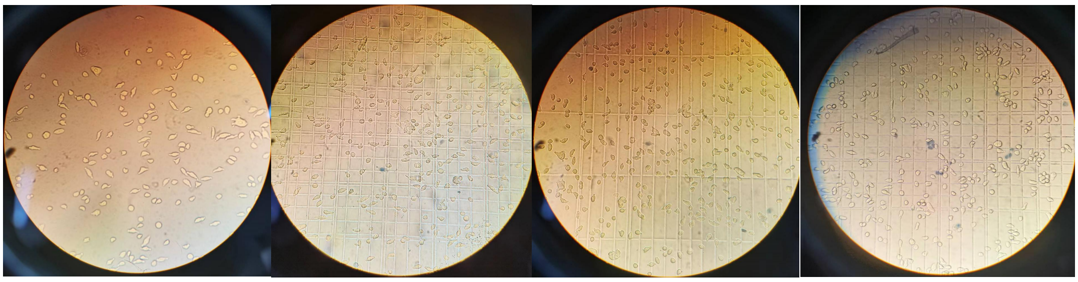

0 h (100%) 2 h (93%) 6 h (98%) 24 h (95%)

Figure S20. Exemplary Trypan blue cell-viability assay of HepG2 after incubation with 0.5 mM BSO for different time, and the viability rate was added in bracket.

3. Supporting Tables

Table S1. The determined overall hydrolysis reaction rate and half lifetime of 0.5 mM ^{19}F-probe (**P1-P3**) in 20 mM PB, pH 7.5 at 298K.

Probe	K_{obs} / h^{-1}	$t_{1/2}$ / h
**P1**	0.038	18.2
**P2**	stable	--
**P3**	6.93	0.15

Table S2. The dissociation constant, K_d, of the adduct formed by **P1** with different small thiol molecules in 20 mM PB, pH 7.5 at 298K.

Thiols	K_d / mM
GSH	0.41 ± 0.013
Hcy	0.38 ± 0.025
Cys	1.72 ± 0.054
NAC	0.33 ± 0.005
BME	0.23 ± 0.007

Table S3. The dissociation constant, K_d, of the adduct formed by **P1** and GSH, **P1**-SG, at different pHs and in different crowding media.

Media	K_d / mM
20 mM PB buffer, pH 5.0	0.38 ± 0.014
20 mM PB buffer, pH 6.0	0.36 ± 0.004
20 mM PB buffer, pH 7.5	0.41 ± 0.013
20 mM PB buffer, pH 8.5	0.41 ± 0.024
30% glycerol, pH 7.5	0.39 ± 0.014
50% glycerol, pH 7.5	0.41 ± 0.017
300 g/L ficoll400, pH 7.5	0.46 ± 0.022
100 g/L lysozyme, pH 7.5	0.32 ± 0.011

Table S4. The cell volume measured by packed cell volume tube.

Cell line	Cell volume (μm^3)
NIH-3T3	3600 ± 400
HEK293T	2550 ± 500
HeLa	2800 ± 100
HepG2	2850 ± 200
A549	4500 ± 400

4. References

[1] Jiang, X.; Yu, Y.; Chen, J.; Zhao, M.; Chen, H.; Song, X.; Matzuk, A. J.; Carroll, S. L.; Tan, X.; Sizovs, A.; Cheng, N.; Wang, M. C.; Wang, J. Quantitative imaging of glutathione in live cells using a reversible reaction-based ratiometric fluorescent probe. *ACS Chem. Biol.* **2015**, *10*, 864−874.

[2] Cui, C. Y.; Li, B.; Cheng, D.; Li, X. Y.; Chen, J. L.; Chen, Y. T.; Su, X. C. Simultaneous quantification of biothiols and deciphering diverse GSH stability in different live cells by ^{19}F tag. *Anal. Chem.* **2022**, *94*, 901-908.

[3] Holtzer, A.; Holtzer, M. F. Use of the van't Hoff relation in determination of the enthalpy of micelle formation. *J. Phys. Chem. C* **1974**, *78*, 1442-1443.
